# Large Oncosome‐Loaded VAPA Promotes Bone‐Tropic Metastasis of Hepatocellular Carcinoma Via Formation of Osteoclastic Pre‐Metastatic Niche

**DOI:** 10.1002/advs.202201974

**Published:** 2022-09-28

**Authors:** Shuxia Zhang, Xinyi Liao, Suwen Chen, Wanying Qian, Man Li, Yingru Xu, Meisongzhu Yang, Xincheng Li, Shuang Mo, Miaoling Tang, Xingui Wu, Yameng Hu, Ziwen Li, Ruyuan Yu, Ainiwaerjiang Abudourousuli, Libing Song, Jun Li

**Affiliations:** ^1^ Program of Cancer Research Key Laboratory of Protein Modification and Degradation and Guangzhou Institute of Oncology Affiliated Guangzhou Women and Children's Hospital School of Basic Medical Sciences Guangzhou Medical University Guangzhou 510623 P. R. China; ^2^ Department of Biochemistry Zhongshan School of Medicine Sun Yat‐sen University Guangzhou 510080 P. R. China; ^3^ State Key Laboratory of Oncology in South China Collaborative Innovation Center for Cancer Medicine Sun Yat‐sen University Cancer Center Guangzhou 510080 P. R. China

**Keywords:** bone metastasis, hepatocellular carcinoma, osteoclastic pre‐metastatic niche, VAMP‐associated protein A (VAPA)

## Abstract

Tumor‐derived extracellular vesicles (EVs) function as critical mediators in selective modulation of the microenvironment of distant organs to generate a pre‐metastatic niche that facilitates organotropic metastasis. Identifying the organ‐specific molecular determinants of EVs can develop potential anti‐metastatic therapeutic targets. In the current study, large oncosomes (LOs), atypically large cancer‐derived EVs, are found to play a crucial role in facilitating bone‐tropic metastasis of hepatocellular carcinoma (HCC) cells by engineering an osteoclastic pre‐metastatic niche and establishing a vicious cycle between the osteoclasts and HCC cells. Transmembrane protein, VAMP‐associated protein A (VAPA), is significantly enriched on LOs surface via direct interaction with LOs marker *α*V‐integrin. VAPA‐enriched LOs‐induced pre‐metastatic education transforms the bone into a fertile milieu, which supports the growth of metastatic HCC cells. Mechanically, LOs‐delivered VAPA integrates to plasma membrane of osteoclasts and directly interacts with and activates neural Wiskott–Aldrich syndrome protein (N‐WASP) via dual mechanisms, consequently resulting in ARP2/3 complex‐mediated reorganization of actin cytoskeleton in osteoclasts and osteoclastogenesis. Importantly, treatment with N‐WASP inhibitor 187‐1‐packaged LOs (LOs/187‐1) dramatically abolishes the inductive effect of VAPA‐enriched LOs on pre‐metastatic niche formation and precludes HCC bone metastasis. These findings reveal a plausible mechanism for bone‐tropism of HCC and can represent a potential strategy to prevent HCC bone metastasis.

## Graphical abstract

Hepatocellular carcinoma (HCC)‐derived VAMP‐associated protein A (VAPA)‐enriched large oncosomes (LOs) facilitate bone metastasis (BM) via engineering an osteoclastic pre‐metastatic niche. LOs‐delivered VAPA directly activates neural Wiskott–Aldrich syndrome protein (N‐WASP) to promote ARP2/3 complex‐mediated actin cytoskeleton remolding in pre‐osteoclasts, consequently resulting in osteoclast differentiation and activation. Importantly, LOs‐packaged N‐WASP inhibitor significantly abrogates the educative effect of VAPA‐enriched LOs on HCC‐BM, which represents a potential strategy to prevent HCC‐BM.

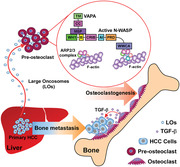

## 1 Introduction

During the evolution from unicellular organisms to multicellular organisms, cells evolved the capability to “communicate” with neighboring and distant cells within the organism, which is termed cell‐to‐cell communication or intercellular communication.^[^ ^1^ ^]^ Through transferring a variety of messages between cells, intercellular communication ensures cooperation between the same and different types of cells and maintains physiological tissue homeostasis. Multiple mechanisms are reported to be involved in intercellular communication, including direct cell‐to‐cell contact and indirect contact through soluble factors, such as cytokines, hormones, or metabolites.^[^ ^2^ ^]^ In addition, the extracellular vesicles (EVs)‐mediated intercellular communication, which is associated with a variety of physiological and pathophysiological contexts, has recently become an emerging area of research.^[^ ^2^, ^3^ ^]^

EVs are a heterogeneous population of membrane‐delineated nano‐vesicles that are released by virtually any cell type into extracellular spaces via both active and passive processes. Based on the mode of biogenesis pathways and sizes, EVs are typically categorized into three major groups: small extracellular vesicles (sEVs, 50–150 nm in diameter), microvesicles (MVs, 100–1000 nm), and apoptotic bodies (500–5000 nm).^[^ ^4^ ^]^ Recently, a new class of gigantic cancer‐derived EVs has been characterized, with atypical size of 1–10 µm in diameter, which is much larger than most typical EV types, thereby referred to as large oncosomes (LOs).^[^ ^5^ ^]^ Importantly, LOs were found to be exclusively shed by cancer cells but not in benign tissues and more abundant in invasive cancer cells and in plasma of patients with metastatic cancer, suggesting that LOs might contribute to cancer metastasis.^[^ ^6^ ^]^ However, the precise mechanism underlying LOs‐mediated metastasis remains largely unknown.

The formation of primary tumors‐mediated microenvironment modulation in distant organs before metastasis is crucial for the subsequent colonization, adaptation, and survival of metastatic cells.^[^ ^7^ ^]^ This supportive metastatic microenvironment in distant organs was named as pre‐metastatic niche (PMN), which plays a curtail role in subsequent colonization and proliferation of tumor cells.^[^ ^8^ ^]^ For instance, it has been reported that HCC cells‐released interleukin (IL)‐11, an important regulatory protein for bone formation, remodeling and resorption, was associated with osteoclast recruitment for formation of bone PMN.^[^ ^9^ ^]^ Zhang et al. reported that HCC‐secreted LGALS3‐induced PMN, via promoting osteoclast fusion and podosome formation, promoted HCC bone metastasis and associated skeletal complications.^[^ ^8c^ ^]^ Furthermore, Zhao et al. found that long noncoding RNA H19‐mediated HCC bone‐metastasis was through induction of osteoclastogenesis, which is a prerequisite for formation of bone PMN, via suppression of osteoprotegerin (OPG).^[^ ^10^ ^]^ Recently, numerous experiments have provided strong evidence that EVs play critical roles in PMN formation via connecting communication between the primary tumor and distant organs by precisely delivering tumor cargos to recipient cells in target organs.^[^ ^11^ ^]^ Therefore, the identification of the key EVs‐cargoed factors that determine organ‐specific patterns would help to develop potential therapeutic interventions in cancer metastasis.

Herein, we reported that hepatocellular carcinoma (HCC)‐derived VAMP‐associated protein A (VAPA)‐enriched LOs significantly promoted HCC bone‐specific metastasis by facilitating an osteoclastic PMN formation through neural Wiskott–Aldrich syndrome protein (N‐WASP)/actin related protein 2 (ARP2)/ARP3 signaling‐mediated fusion and activation of osteoclasts. Importantly, treatment with anti‐VAPA antibodies or the 187‐1 (LOs/187‐1) markedly prevented VAPA‐enriched LOs‐induced bone pre‐metastatic niche formation and HCC bone‐metastasis. Therefore, our results revealed a plausible mechanism for bone‐tropism of HCC and might represent a potential strategy to treat HCC bone metastasis.

## 2 Results

### 2.1 Bone‐Metastatic HCC Cells‐Derived LOs Induce Pre‐Metastatic Bone Lesions

**Figure 1:**
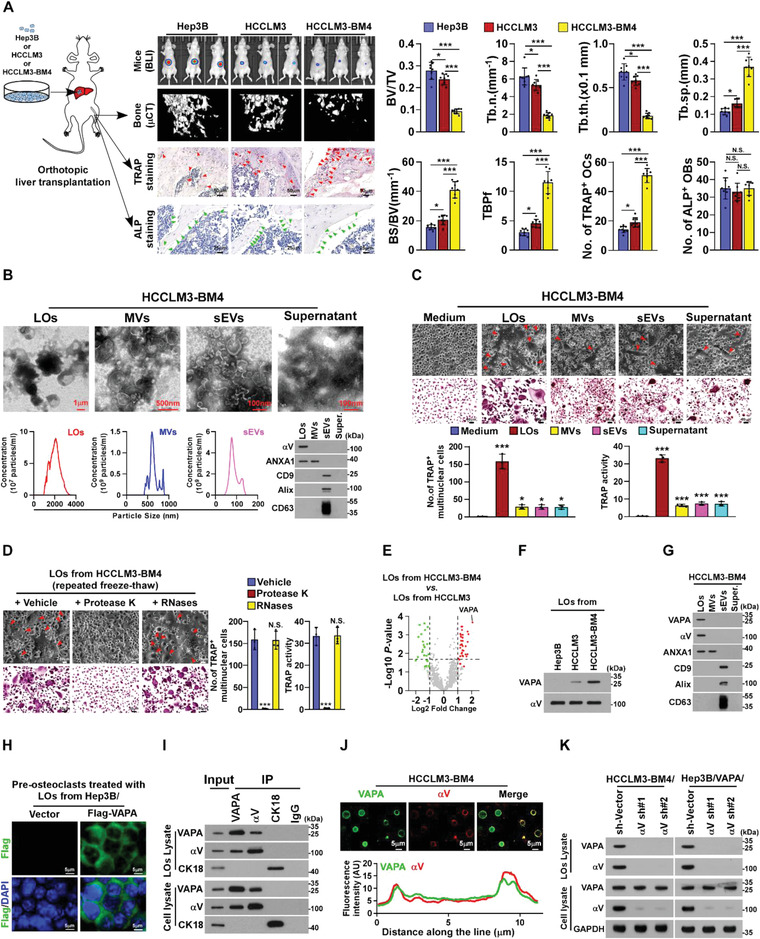
Large oncosomes‐loaded VAPA protein promotes osteoclastogenesis. A) Left: Schematic representation of the establishment of a mouse orthotopic liver cancer model. Human hepatocellular carcinoma Hep3B or HCCLM3 or HCCLM3‐BM4 cells were transplanted into the livers of nude mice, and then, the mice were monitored for liver xenografts tumor growth and the bone microarchitecture. Middle: BLI images of liver xenografts tumor (upper) and µCT images of bone trabecular sections (middle), and histological (TRAP and ALP) images (lower) of TRAP^+^‐osteoclasts/ALP^+^‐osteoblasts along the bone interface from representative mice (*n* = 8 per group). Scale bar, 50 µm. Right: quantification of the indicated bone parameters and number of TRAP^+^‐osteoclasts/ALP^+^‐osteoblasts from representative mice (*n* = 8 per group). BV/TV, bone/tissue volume ratio; BS/TV, bone surface/tissue volume ratio; Tb. *n*, trabecular number; Tb. sp., trabecular separation; Tb. th., trabecular thickness; TBPf, trabecular bone pattern factor. B) Upper: representative transmission electron microscopy (TEM) images of large oncosomes (LOs), microvesicles (MVs), small extracellular vesicles (sEVs), and supernatant from HCCLM3‐BM4 cells. Scale bar, 1 µm or 100 nm or 500 nm. Lower‐left: qNano analysis of LOs, MVs, and sEVs derived from bone metastatic HCC cells using NP2000 nm, NP800 nm, and NP100 nm membrane pores, respectively. Lower‐right: IB analysis of expression of *α*V‐integrin, ANXA1, CD9, Alix and CD63 in LOs, MVs, sEVs, and supernatant. C) Upper: phase contrast and TRAP staining images of osteoclast differentiation assays under the indicated treatments. Scale bar, 20 µm or 50 µm. Lower: quantification of the number of TRAP^+^ multinuclear cells and TRAP activity from experiments in the upper panel. D) Left: phase contrast (upper) and TRAP staining images (middle) of pre‐osteoclasts treated with HCCLM3‐BM4 cells‐derived LOs after five repeated freeze–thaw cycles and plus vehicle, or protease K (20 mg mL^−1^) or RNase (0.02 mg mL^−1^). Scale bar, 20 µm or 50 µm. Right: quantification of the number of TRAP^+^ multinuclear cells and TRAP activity from the experiment in the left panel. E) Volcano plot analysis revealed that VAPA levels were mostly elevated in LOs/HCCLM3‐BM4 compared to LOs/HCCLM3. F) IB analysis of VAPA levels in LOs from Hep3B, HCCLM3, and HCCLM3‐BM4 cells. *α*V‐integrin served as loading control. G) IB analysis of expression of VAPA, *α*V‐integrin, ANXA1, CD9, Alix, and CD63 in LOs, MVs, sEVs, and supernatant from HCCLM3‐BM4 cells. H) IF staining using anti‐Flag antibody in pre‐osteoclasts treated with LOs derived from Hep3B/Vector or Hep3B/Flag‐VAPA cells. Scale bar, 5 µm. I) Co‐IP/IB analyses of interaction of endogenous VAPA with *α*V‐integrin and CK‐18 in the HCCLM3‐BM4 cells and LOs derived from HCCLM3‐BM4 cells. J) IF staining analysis of LOs derived from HCCLM3‐BM4 cells using anti‐VAPA and anti‐*α*V‐integrin antibody (upper) and line scans analysis of signal intensity of VAPA and *α*V‐integrin in LOs (lower). Scale bar, 5 µm. K) IB analysis of levels of *α*V‐integrin and VAPA in the indicated control or *α*V‐integrin‐silenced cells and LOs. GAPDH served as the loading control. Each error bar represents the mean ± SD of three independent experiments. Significant differences were determined by one‐way ANOVA with Tukey's multiple comparison test (A,C,D). * *P* < 0.05, *** *P* < 0.001, and N.S. > 0.05.

To identify the key factor(s) contributing to bone pre‐metastatic niche formation, an orthotopic liver cancer model was established using non‐metastatic Hep3B hepatoma cells, or highly metastatic HCCLM3 cells, or bone‐metastatic HCCLM3‐BM4 cells^[^ ^8^, ^12^ ^]^ (**Figure** **1**A). Micro‐computed tomography (µCT) was employed to monitor the alteration of the bone microarchitecture and bioluminescence imaging (BLI) was employed to analyze the tumor growth and metastasis. Prominently, even without distant metastasis, we observed drastic changes in the bone microarchitecture, as indicated by significantly increased trabecular separation and trabecular bone pattern factor, and decreased trabecular volume, trabecular number, and trabecular thickness, in the HCCLM3‐BM4/mice, compared with the HCCLM3/mice and Hep3B/mice (Figure 1A). All these abovementioned altered factors suggested that HCCLM3‐BM4‐formed tumors might induce typical osteolytic bone lesions. In line with this hypothesis, histological analysis revealed that the number of TRAP^+^‐osteoclasts, but no obvious alteration of alkaline phosphatase (ALP)^+^‐osteoblasts, was significantly increased along the trabecular bone surfaces in HCCLM3‐BM4/mice (Figure 1A). Taken together, these results suggested that HCCLM3‐BM4 cells in situ could induce the formation of an osteolytic bone pre‐metastatic niche.

To determine the key factor (s) that contribute to bone pre‐metastatic niche formation, the differential centrifugation method, followed by density gradient purification, was employed to isolate the LOs, MVs, sEVs, and supernatant from the conditioned medium (CM) of Hep3B, HCCLM3, and HCCLM3‐BM4 cells, which were further confirmed by transmission electron microscopy (TEM), a qNano particle analyzer, and immunoblotting (IB) analysis using the corresponding markers (Figure 1B). Strikingly, treatment with LOs/HCCLM3‐BM4 exhibited the most inductive effect on osteoclastogenesis, as indicated by the increased number of TRAP^+^‐multinuclear osteoclasts and TRAP enzymatic activity, compared with those of the other isolated components (Figure 1C; Figure S1A–C, Supporting Information). These results suggest that LOs derived from HCC metastatic cells play a key role in inducing the pre‐metastatic niche in bone.

### 2.2 VAPA is Enriched in LOs Derived From Bone‐Metastatic HCC Cells

Osteoclastogenesis assays revealed that the inductive effect of LOs/HCCLM3‐BM4 on osteoclastogenesis was significantly abolished by treatment with protease K but not with RNase, which suggested that the proteins loaded in LOs were essential for LOs‐induced osteoclastogenesis (Figure 1D). We then performed the mass spectrometry‐based proteomics to analyze the differential abundance of protein in LOs isolated from HCCLM3 and HCCLM3‐BM4 cells. Protein profiling analysis revealed a total of 61 dysregulated proteins, including 36 upregulated proteins and 25 downregulated proteins, in LOs derived from HCCLM3‐BM4 cells compared with LOs from HCCLM3‐parental cells as showed in Table S1, Supporting Information. As exemplified in Figure 1E; Table S1, Supporting Information, the abundance of VAPA, a type IV membrane protein that is present in the cellular vesicle and plasma membrane,^[^ ^13^ ^]^ was mostly elevated in LOs/HCCLM3‐BM4 compared with that in LOs/HCCLM3. Furthermore, IB analysis showed that VAPA expression was nearly undetectable in LOs/Hep3B but became higher in LOs/HCCLM3 and was markedly elevated in LOs/HCCLM3‐BM4 (Figure 1F). Interestingly, IB and immunofluorescence staining (IF) analyses indicated that VAPA protein was only localized in LOs but not in other components (Figure 1G; Figure S1D, Supporting Information). Moreover, we found that VAPA was expressed on the LOs surface and treatment with LOs derived from Hep3B/Flag‐VAPA cells significantly increased VAPA expression in the membrane of pre‐osteoclasts (Figure 1H; Figure S1E,F, Supporting Information). Taken together, these results suggest that the VAPA is enriched in LOs of bone‐metastatic cells.

### 2.3 VAPA Interacts With LOs marker *α*V‐integrin and is Sorted Into LOs Surface

We further investigate the mechanism underlying the VAPA enriched on LO's surface. Previously, multiple proteins, such as including cytokeratin 18 (CK18) and *α*V‐integrin, were identified as LOs marker.^[^ ^6^, ^14^ ^]^ Co‐immunoprecipitation (co‐IP) assays showed that VAPA could interact with *α*V‐integrin, but not CK18, in both HCC cells and HCC cells‐derived LOs (Figure 1I; Figure S2A, Supporting Information). This was further confirmed by the IF staining that VAPA co‐localized with *α*V‐integrin on the LOs surface and peak staining intensities of VAPA and *α*V‐integrin tended to be consistent with each other (Figure 1J). Moreover, far‐western blotting analysis indicated that VAPA could directly interact with *α*V‐integrin (Figure S2B, Supporting Information). Analysis using the ClusPro server4‐8 (https://cluspro.org) predicted that the residues 29‐126 in the N‐terminus of VAPA docked with residues 989‐1045 in the GFFKR region of *α*V‐integrin (Figure S2C, Supporting Information). This automated prediction of VAPA/*α*V‐integrin interaction was further confirmed by co‐IP assays using serially truncated VAPA fragments and *α*V‐integrin, which showed that the MSP region of VAPA was required for *α*V‐integrin interaction (Figure S2D, Supporting Information).

Importantly, silencing *α*V‐integrin significantly reduced the VAPA level in HCC cells‐derived LOs but has no impact on VAPA expression in HCC cells, which indicated that VAPA was recruited by *α*V‐integrin into the LOs surface (Figure 1K). Consistently, silencing *α*V‐integrin in the HCC cells significantly abolished the inductive effect of HCCLM3‐BM4 cells‐derived LOs on the formation of TRAP^+^‐multinuclear osteoclasts and TRAP enzymatic activity (Figure S2E, Supporting Information). Taken together, these results indicate that the LOs marker *α*V‐integrin associates with and sorts VAPA into LOs.

### 2.4 LOs‐Loaded VAPA Protein Promotes Osteoclastogenesis

**Figure 2:**
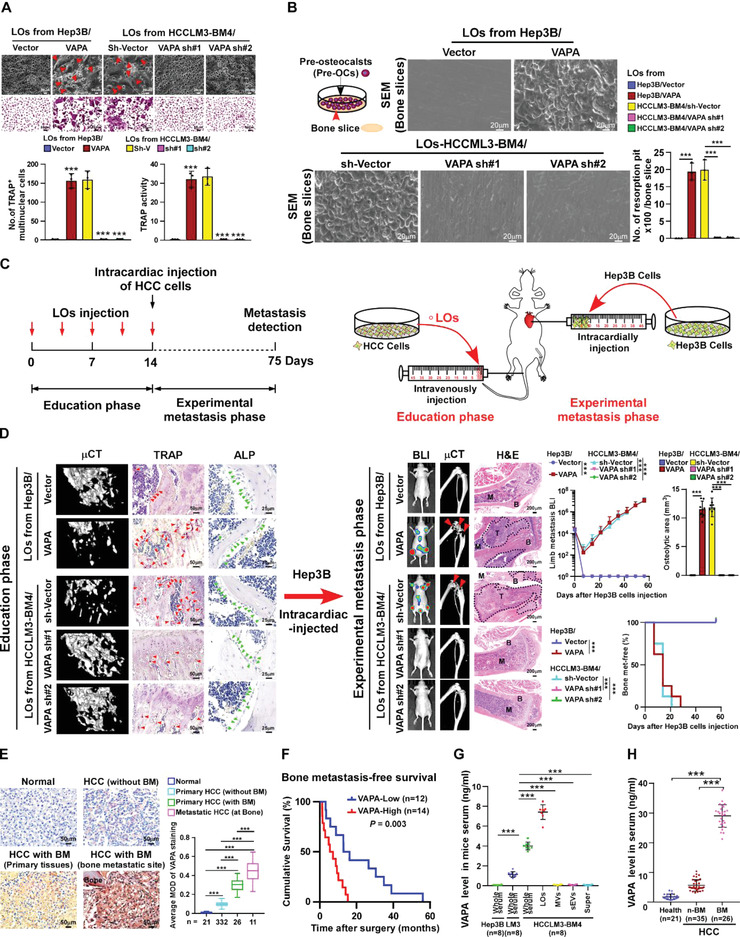
VAPA‐loaded LOs induce osteolytic bone destruction and a vicious cycle in vitro and induce bone pre‐metastatic niche formation in vivo. A) Upper and middle: phase contrast (upper) and TRAP staining (middle) images of pre‐osteoclasts treated with the indicated LOs. Scale bar, 20 µm or 50 µm. Lower: quantification of number of TRAP^+^ multinuclear cells and TRAP activity from experiment in the upper and middle panel. B) Bone resorption assays of pre‐osteoclasts cultured onto the bone slices under the indicated treatments. Then, bone slices were fixed for scanning electron microscopy (SEM) (left) and quantification of the number of resorption pits per bone slice (right). Scale bar, 20 µm. C) Schematic illustration of mouse model of the education phase (left) and experimental metastasis phase in vivo (right). D) Left: µCT images of bone trabecular section and histological (TRAP and ALP) images of TRAP^+^‐osteoclasts/ALP^+^‐osteoblasts along the bone interface from the indicated mice in education phase (*n* = 8 per group). Scale bar, 50 µm or 25 µm. Middle: BLI and µCT, and histological (H&E) images of bone tumor and lesions from the indicated mice in the experimental metastasis phase. Scale bar, 200 µm. Right: normalized BLI signals of bone metastases, quantification of the µCT osteolytic lesion area, and bone metastasis‐free survival curve of the indicated mice from the experimental metastasis phase (*n* = 8 per group). E) Representative images (left) and quantification (right) of VAPA expression in normal liver tissue (*n* = 21), HCC tissues without bone metastasis (*n* = 332), primary HCC tissues with bone metastasis (*n* = 26), and HCC tissues in bone metastatic site (*n* = 11) (left panel). Scale bar, 50 µm. F) Kaplan–Meier analysis of bone metastasis‐free survival curves in HCC‐BM with low versus high expression of VAPA (*n* = 26; *P* = 0.003, log‐rank test). G) ELISA analysis of VAPA levels in the whole serum of Hep3B/mice and HCCLM3/mice and in the whole serum and LOs, MVs, sEVs, and supernatant derived from HCCLM3‐BM4/mice (*n* = 8). H) ELISA analysis of serum VAPA levels in healthy donors (*n* = 21), HCC patients without bone metastasis (*n*‐BM, *n* = 35), and HCC patients with bone metastasis (BM, *n* = 26). Each error bar represents the mean ± SD of three independent experiments. Significant differences were determined by one‐way ANOVA with Tukey's multiple comparison test (A–H). *** *P* < 0.001.

Prominently, the LOs derived from VAPA‐overexpressing Hep3B cells significantly increased the number of TRAP^+^‐multinuclear osteoclasts and TRAP enzymatic activity (**Figure** **2**A), whereas the inductive effect of LOs from HCCLM3‐BM4 cells on osteoclastogenesis was dramatically abolished by *VAPA* silencing (Figure S3A, Supporting Information; Figure 2A). Meanwhile, we found that treatment with VAPA‐neutralizing antibodies also showed dramatic inhibiting effect on VAPA‐enriched LOs‐induced osteoclastogenesis (Figure S3B, Supporting Information). However, we did not observe an obvious effect of HCC cells‐derived LOs on the differentiation of pre‐osteoblast MC3T3‐E1 cells, as indicated by an unaltered number of ALP^+^‐osteoblasts and the relative RANKL/OPG ratio (Figure S3C,D, Supporting Information). Taken together, these results suggest LOs‐loaded VAPA might contribute to formation of osteoclastic pre‐metastatic niche.

### 2.5 VAPA is Essential for LOs‐Induced Osteolytic Bone Destruction and the Vicious Cycle

To further confirm the inductive effect of VAPA‐enriched LOs on osteoclastogenesis, a bone resorption assay was performed using human pre‐osteoclasts. As exemplified in Figure 2B, scanning electron microscopy (SEM) analysis revealed that the surface of the bone slice was severely eroded by pre‐osteoclasts treated with LOs from VAPA‐overexpressing Hep3B cells or HCCLM3‐BM4 cells, as indicated by the increased formation of resorption pits. However, silencing VAPA in HCCLM3‐BM4 cells drastically abolished the eroded effect of LOs‐HCCLM3‐BM4‐treated pre‐osteoclasts. Moreover, we found that treatment with VAPA‐neutralizing antibodies also showed dramatic inhibiting effect on VAPA‐enriched LOs‐induced osteolytic bone destruction (Figure S3E, Supporting Information). Therefore, these results indicate that VAPA‐enriched LOs induce the resorption activity of osteoclasts, eliciting osteolytic bone destruction.

It has been reported that resorption of the bone matrix by activated osteoclasts could release various bone matrix‐bound factors, such as transforming growth factor beta (TGF‐*β*), which facilitates the seeding and expansion of metastatic tumor cells in bone, thus forming a “vicious cycle”.^[^ ^15^ ^]^ We then further examined the TGF‐*β* level released from LOs‐treated bone slice and the effect on the proliferation of HCC cells. As exemplified in Figure S3F, Supporting Information, accompanied by severely eroded bone slice by pre‐osteoclasts treated with VAPA‐overexpressing Hep3B cells‐derived LOs, the bone slice‐released TGF‐*β* was significantly elevated, which consequently resulted in higher growth rate of Hep3B cells. However, silencing VAPA or treatment with VAPA‐neutralizing antibodies drastically abolished the inductive effect of the HCCLM3‐BM4 cells‐derived LOs on bone slice‐released TGF‐*β* and the proliferation of Hep3B cells (Figure S3F,G, Supporting Information). Taken together, these results suggest that VAPA‐enriched LOs could induce osteolytic bone destruction and a vicious cycle of bone metastasis.

### 2.6 Education by VAPA‐Enriched LOs Induces Bone Pre‐Metastatic Niche In Vivo

Next, an in vivo experimental model was employed to examine the effect of VAPA‐enriched LOs on pre‐metastatic niche formation and bone metastasis. Mice were first educated with VAPA‐deregulated HCC‐derived LOs for 14 days (the education phase), and were then injected intracardially with luciferase‐expressing Hep3B cells (experimental metastasis phase) (Figure 2C). Strikingly, the mice educated by LOs‐Hep3B/VAPA displayed significant alteration in their bone microarchitecture, as indicated by enlarged osteolysis onsets and decreased trabecular volume/number/thickness but increased trabecular separation/bone pattern factor. Compared with the control LOs‐educated mice, education with LOs derived from VAPA silenced‐HCCLM3‐BM4 cells only resulted in tiny osteolytic lesions and alterations to the bone microarchitecture in the mice (Figure 2D; Figure S4A, Supporting Information). Consistently, the number of TRAP^+^‐osteoclasts, but not ALP^+^‐osteoblasts, along the trabecular bone surfaces was increased in the mice educated by VAPA‐enriched LOs but decreased in the VAPA‐silenced LOs‐treated mice (Figure 2D). Meanwhile, we also found that the expression of multiple immune components, including CXCL12, CCL2, and CCL4 that was previously reported to be upregulated in bone pre‐metastatic niche,^[^ ^16^ ^]^ was dramatically increased in the bone marrow supernatant in femur and tibial of mice pre‐educated by Hep3B/VAPA‐derived LOs (Figure S4B, Supporting Information). Taken together, these results suggest that education by VAPA‐enriched LOs induces bone pre‐metastatic niche formation in vivo.

Consistent with the educating effect of LOs on bone pre‐metastatic niche formation, further in vivo experimental metastatic assays showed that pre‐education by VAPA‐overexpressing Hep3B cells‐ or HCCLM3‐BM4 cell‐derived LOs significantly enhanced the bone‐metastatic capability of non‐metastatic Hep3B cells, as indicated by severe osteolytic bone lesion and earlier bone metastasis, which resulted in significantly shorter bone‐metastasis‐free survival (Figure 2D). Importantly, we only found strong metastatic signals in bone, including hindlimb bone, and forelimb bone and spine, but not in brain, lung, heart, spleen, liver, intestine and kidney, monitored by bioluminescence signal analysis (Figure S4C, Supporting Information). Furthermore, the promotive effect of LOs/HCCLM3‐BM4 on the bone‐metastatic potential of Hep3B cells was abolished by VAPA downregulation or VAPA‐neutralizing antibody treatment, consequently leading to longer survival of bone‐metastatic mice (Figure 2D; Figure S4D,E Supporting Information). These results provided further evidence that VAPA‐enriched LOs play a crucial role in facilitating HCC bone‐tropic metastasis.

Moreover, an in vivo experimental model using murine Hepa1‐6 hepatoma cell line and immunocompetent BALB/c mice was established to further examine the effect of VAPA‐enriched LOs on pre‐metastatic niche formation and bone metastasis. The BALB/c mice were first pre‐educated with LOs derived from Hepa1‐6/Vector cells or Hepa1‐6/VAPA cells for 14 days (the education phase), and then, were injected intracardially with luciferase‐expressing Hep1‐6 cells (experimental metastasis phase) (Figure S5A,B, Supporting Information). As shown in Figure S5C, Supporting Information, the mice pre‐educated with LOs derived from Hepa1‐6/VAPA cells displayed significant alterations in their bone microarchitecture, as indicated by decreased trabecular volume/number/thickness but increased trabecular separation/bone pattern factor, compared with the control mice pre‐educated with LOs derived from Hepa1‐6/vector cells. Meanwhile, we found that the number of TRAP^+^‐osteoclasts along the trabecular bone surfaces was significantly increased in the mice pre‐educated by VAPA‐enriched LOs compared with control mice (Figure S5C, Supporting Information). These results provided further evidence that pre‐education by VAPA‐enriched LOs induced bone pre‐metastatic niche formation in vivo. Consistent with the educating effect of LOs on bone pre‐metastatic niche formation, in vivo experimental metastatic assays revealed that pre‐education by Hepa1‐6/VAPA‐derived LOs significantly enhanced the bone‐metastatic ability of Hepa1‐6 cells, as indicated by severe osteolytic bone lesion and earlier bone metastasis, which resulted in significantly shorter bone‐metastasis‐free survival (Figure S5C, Supporting Information). These results further supported the notion that VAPA‐enriched LOs promoted HCC bone‐tropic metastasis via formation of osteoclastic pre‐metastatic niche.

### 2.7 The Serum VAPA Level Correlates With Bone Metastasis in HCC

Next, we further examined the clinical correlation of VAPA expression with HCC bone metastasis. As shown in Figure 2E, VAPA expression was rarely detected in normal liver tissues and remained low in primary non‐bone‐metastatic HCC tissues, while it was markedly increased in primary HCC‐BM tissues and further elevated in HCC bone‐metastasis tissue. Statistical analysis revealed that the HCC patients with high VAPA‐expression exhibited significantly shorter bone‐metastasis‐free survival compared with HCC patients with low VAPA‐expression and VAPA expression could be recognized as an independent prognostic factor for survival in patients with HCC bone‐metastasis (*p* = 0.003, Figure 2F; Tables S2–S4, Supporting Information), which provided the further evidence to link VAPA overexpression with HCC bone‐metastasis.

The correlation of the serum VAPA level with bone metastasis was then examined in HCC. ELISA analysis showed that the serum level of VAPA was undetectable in Hep3B/mice and became higher in HCCLM3‐P/mice but was markedly elevated in HCCLM3‐BM4/mice, suggesting that the serum VAPA level was increased prior to HCC bone metastasis. Importantly, the expression of VAPA protein was hardly detected in sEVs, MVs, and supernatant but was dramatically elevated in LOs isolated from HCCLM3‐BM4/mice serum, where the LOs‐loaded VAPA level was nearly same as the serum VAPA level (Figure 2G). These results indicated that the VAPA protein in serum was mainly enriched in LOs. Moreover, we found that the serum level of VAPA, which was at a very low level in healthy serum, was significantly higher in HCC‐BM patients than that in HCC patients without bone‐metastasis (Figure 2H). Taken together, these results suggest that the serum VAPA level could be used as a potential biomarker for HCC‐BM.

### 2.8 LOs‐Loaded VAPA Induces Osteoclast Actin Cytoskeletal Remodeling

**Figure 3:**
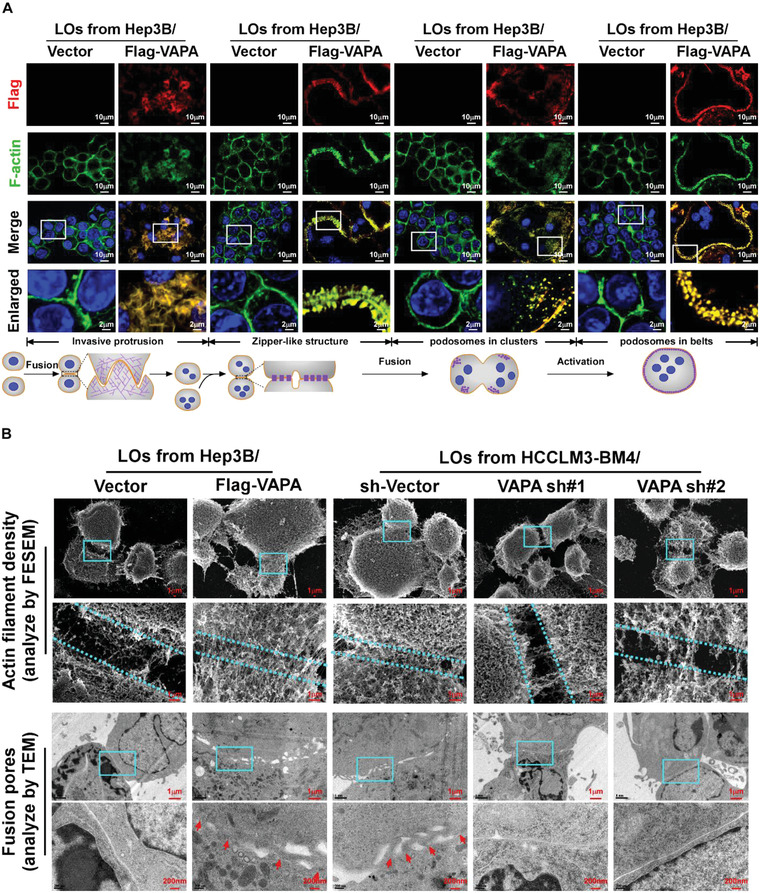
LOs‐loaded VAPA induces osteoclast actin cytoskeletal remodeling. A) Osteoclast precursor cells were treated with LOs derived from Hep3B/Vector or Hep3B/Flag‐VAPA cells, followed by IF staining using anti‐Flag and anti‐F‐actin antibodies during osteoclastogenesis. Scale bar, 10 µm or 2 µm. B) Upper: Representative FESEM image of the actin filament density at the surface of two fused pre‐osteoclasts treated with LOs from the indicated cells. Lower: Representative TEM image of the membrane fusion between the two fused pre‐osteoclasts treated with LOs from the indicated cells. Scale bar, 1 µm.

Interestingly, immunostaining revealed that, the LOs‐loaded VAPA protein in osteoclasts was co‐localized with cytoskeletal F‐actin, which forms the branched actin network (BAN), zipper‐like actin superstructures (ZLS), and podosome during osteoclastogenesis (**Figure** **3**A). These results suggested that the VAPA protein loaded on HCC cell‐derived LOs might be involved in actin cytoskeletal reorganization in osteoclasts. Therefore, these results indicate that LOs‐loaded VAPA is involved in dynamic remodeling of the actin cytoskeleton in osteoclasts.

The effect of LOs‐loaded VAPA protein on the organization of the actin cytoskeleton in osteoclasts was further examined using field emission scanning electron microscope (FESEM) and transmission electron microscopy (TEM). As exemplified in Figure 3B, VAPA‐enriched LOs treatment resulted in a significantly increased actin filament density at the contact point of two fused pre‐osteoclasts and an apparent ZLS between the broad contact surfaces of fused multinucleated cells. However, silencing VAPA dramatically abrogated the inductive effect of LOs derived from VAPA‐overexpressing cells on the actin‐cytoskeletal organization (Figure 3B). Therefore, these results suggest that VAPA‐mediated osteoclastogenesis acts via remodeling of the actin cytoskeleton.

### 2.9 LOs‐Loaded VAPA Promotes F‐Actin Nucleation Via Activation of the ARP2/3 Complex

**Figure 4:**
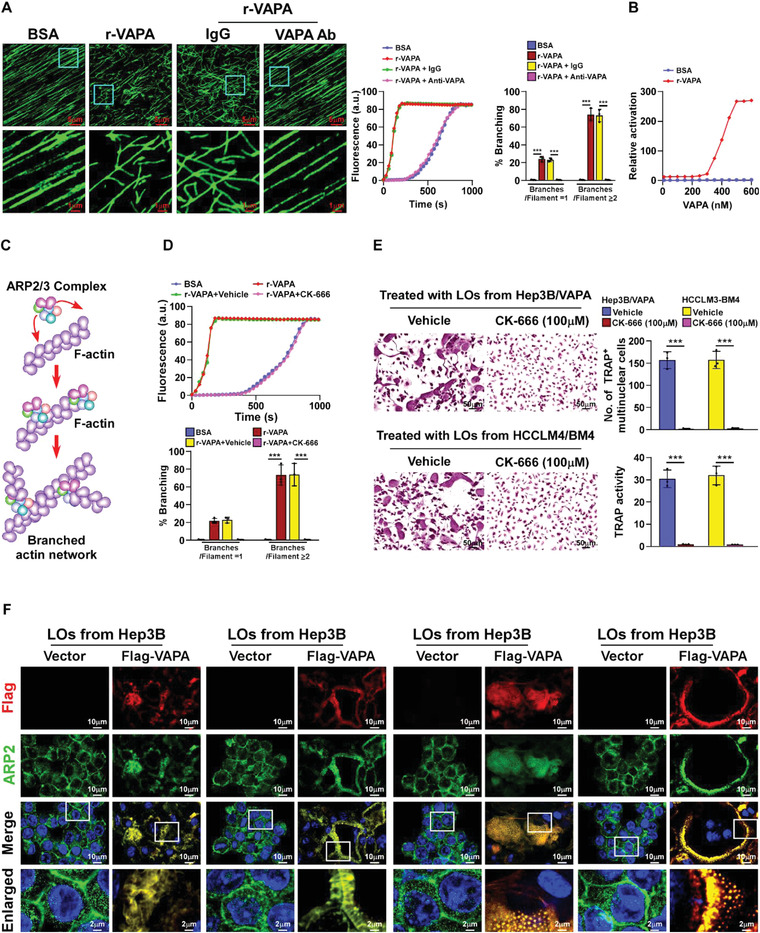
VAPA promotes osteoclastogenesis via ARP2/3 induced‐F‐actin nucleation. A) Left: immunofluorescence images of Alexa488‐actin polymerization treated with BSA, recombinant VAPA protein, recombinant VAPA protein plus IgG or anti‐VAPA antibodies. Conditions were as follows: 1.5 µm 10% Alexa488‐labelled actin monomer, 80nM ARP2/3 complex; 100 nm N‐WASP, 500 nm recombinant VAPA, and 500 nm IgG or anti‐VAPA antibody. Scale bar, 5 µm (upper) or 1 µm (lower). Middle: quantification of fluorescence of Alexa488‐actin polymerization assays in the left panel. Right: branching was quantified as follows: % branching = (number of branched filaments/number of total filaments) × 100. Quantification of percent branching was subcategorized into the percent of total filaments with one branch and the percent of total filaments with two or more branches. B) Dose‐response curves showing the variation in the maximum rate of filament elongation as a function of increasing concentrations of VAPA in the presence of ARP2/3 complex (80 nm) and N‐WASP (400 nm). C) Schematic representation of ARP2/3 complex mediated F‐actin nucleation. D) Upper: quantification of fluorescence of Alexa488‐actin polymerization assays, which were initiated with VAPA (500 nm) in the presence of vehicle or ARP2/3 complex inhibitor CK‐666 (100 µm). Lower: quantitative analysis of filament branching after the initiation of polymerization. E) Left: TRAP staining images of pre‐osteoclasts treated with Hep3B/VAPA‐ or HCCLM3‐BM4 cells‐derived LOs plus vehicle or CK‐666 (100 µm). Scale bar, 50 µm. Right: quantification of number of TRAP^+^ multinuclear cells and TRAP activity from experiment in the left panel. F) IF staining using anti‐Flag and anti‐ARP2 antibodies in pre‐osteoclasts treated with Hep3B/Vector cells‐ or Hep3B/Flag‐VAPA cells‐derived LOs. Scale bar, 10 µm or 2 µm. Each error bar represents the mean ± SD of three independent experiments. Significant differences were determined by one‐way ANOVA with Tukey's multiple comparison test (A,D,E). *** *P* < 0.001.

To explore the mechanism underlying VAPA‐mediated actin cytoskeletal remodeling, a cell‐free actin nucleation assay using recombinant VAPA was performed. As exemplified in **Figure** **4**A,B, addition of VAPA protein significantly increased the formation of the branched actin meshwork in a dose‐dependent manner. This is because activation of ARP2/3 complex plays a vital role in the generation of the BAN^[^ ^17^ ^]^ (Figure 4C), which prompted us to examine whether VAPA protein activated ARP2/3 complex in osteoclasts. We found that treatment with CK‐666, a cell‐permeable selective inhibitor of ARP2/3 complex, drastically abolished VAPA‐mediated actin assembly (Figure 4D), consequently resulting in inhibition of VAPA‐enriched LOs‐induced osteoclastogenesis, as indicated by the reduction of TRAP^+^‐multinuclear osteoclasts and TRAP enzymatic activity (Figure 4E; Figure S6A, Supporting Information). Therefore, these results suggest that activation of ARP2/3 complex is essential for VAPA‐induced actin cytoskeleton reorganization and osteoclastogenesis.

### 2.10 N‐WASP Contributes to VAPA‐Mediated ARP2/3 Activation

**Figure 5:**
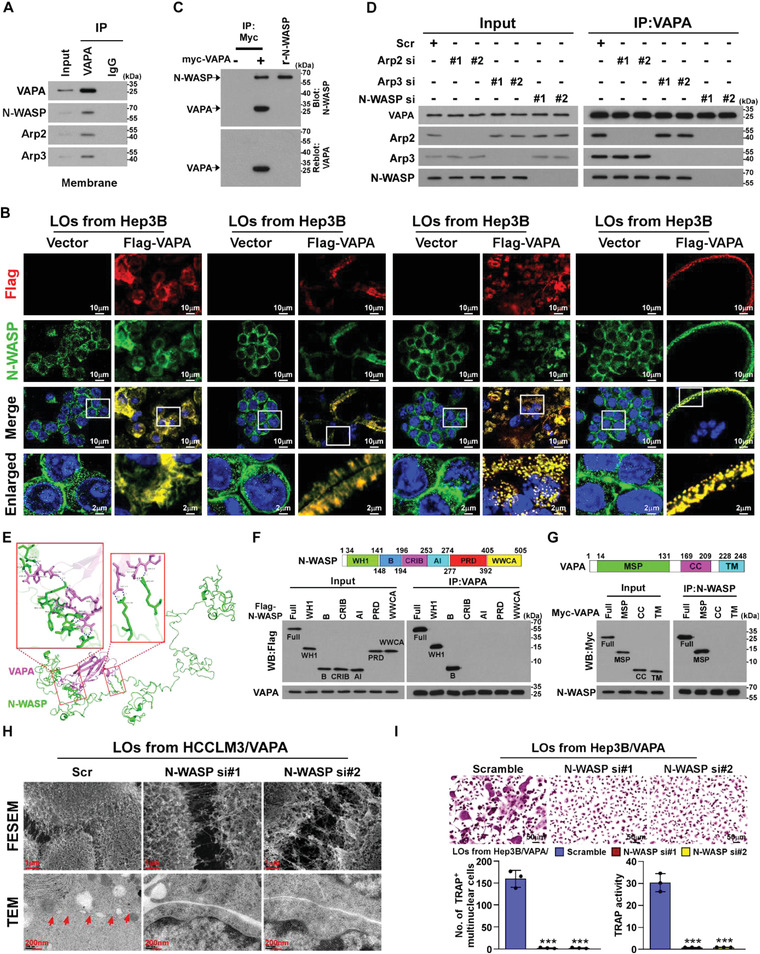
N‐WASP contributes to VAPA‐mediated ARP2/3 activation. A) Co‐IP assays using anti‐VAPA or anti‐IgG antibodies in the membrane of pre‐osteoclasts treated with LOs derived from HCCLM3‐BM4 cells and IB analysis of expression of the levels of VAPA, N‐WASP, ARP2, and ARP3. B) IF staining using anti‐Flag and anti‐N‐WASP antibodies in pre‐osteoclasts treated with LOs derived from Hep3B/Vector cells or Hep3B/FLAG/VAPA cells. Scale bar, 10 µm or 2 µm. C) Far‐western blotting analysis was performed using anti‐myc antibody‐immunoprecipitated proteins and detected using anti‐N‐WASP antibody and then re‐blotted with anti‐VAPA antibody. Recombinant N‐WASP served as the control. D) Co‐IP assays using anti‐VAPA antibody in the indicated cells and IB analysis of levels of VAPA, N‐WASP, ARP2, and ARP3. E) 3D structure of the interaction region between N‐WASP and VAPA. F) Upper: schematic illustration of N‐WASP protein structure, including an N‐terminal WASP‐homology‐1 (WH1), a basic (B), a CDC42/Rac‐interactive binding (CRIB), auto‐inhibitory (AI), a proline‐rich domain (PRD), and a VCA/WWCA domain. Lower: co‐IP assays were performed using anti‐VAPA antibody in the indicated cells. G) Upper: schematic illustration of VAPA protein structure, including major sperm protein (MSP), central coiled‐coil (CC), and transmembrane domain (TMD) domain. Lower: co‐IP assays were performed using anti‐N‐WASP antibody in the indicated cells. H) Upper: representative FESEM imaging of the actin filament density at the surface of two fusing N‐WASP‐silenced pre‐osteoclasts treated with LOs from the indicated cells. Lower: representative TEM imaging of the membrane fusion between the two fused N‐WASP‐silenced pre‐osteoclasts treated with LOs from the indicated cells. I) Upper: TRAP staining images of N‐WASP‐silenced pre‐osteoclasts treated with LOs from the indicated cells. Scale bar, 50 µm. Lower: quantification of number of TRAP^+^ multinuclear cells and TRAP activity from experiment in the upper panel. Each error bar represents the mean ± SD of three independent experiments. Significant differences were determined by one‐way ANOVA with Tukey's multiple comparison test (I). *** *P* < 0.001.

Consistent with the immunostaining results showing that LOs‐loaded VAPA was co‐localized with ARP3 at the BAN, ZLS, and podosomes in the membrane of osteoclasts (Figure 4F), co‐immunoprecipitation (co‐IP) analysis using anti‐Flag antibodies revealed that VAPA formed a complex with ARP2 and ARP3 protein in the membrane of osteoclasts treated with VAPA‐enriched LOs (**Figure** **5**A). However, far‐western blotting assay showed that VAPA protein did not directly interact with the ARP2/ARP3 complex (Figure S7A, Supporting Information), indicating that other protein(s) are involved in formation of the VAPA/ARP2/ARP3 complex.

VAPA interacted and co‐localized with ARP2/ARP3 in the osteoclast membrane (Figures 4F and 5A), which suggested that the VAPA‐associated ARP2/ARP3 complex was in an activated form. It has been reported that the conserved VCA domains of N‐WASP protein activate the ARP2/3 complex by inducing conformational changes and delivering the actin monomer to the filament.^[^ ^18^ ^]^ Interestingly, co‐IP and IF assays showed that LOs‐loaded VAPA also interacted and co‐localized with N‐WASP at the BAN, ZLS, and podosomes in the membrane of osteoclasts (Figure 5A,B). Far‐western blotting assay further demonstrated that VAPA was bound directly to N‐WASP (Figure 5C). Importantly, silencing N‐WASP completely abrogated the association of VAPA with the ARP2/ARP3 complex but knockdown of either ARP2 or ARP3 did not impact the interaction of VAPA with N‐WASP (Figure 5D). These results suggested that N‐WASP is involved in the complex formation of VAPA with ARP2/ARP3.

To further determine the interaction region of N‐WASP with VAPA, ClusPro server4‐8 (https://cluspro.org) was performed for molecular‐docking simulations. As exemplified in Figure 5E, the Ramachandran plot analysis suggested that the 3D structure of N‐WASP protein, from residue 25 to 196 within the WH1 and B domain, and VAPA protein, from residue 57 to 110 within the MSP domain, was automatically docked with each other. This automated prediction of N‐WASP/VAPA interaction was further confirmed by co‐IP assays using serially truncated N‐WASP and VAPA fragments (Figure 5F,G).

We then examined the contribution of N‐WASP to VAPA‐mediated actin cytoskeletal remodeling and osteoclastogenesis. As exemplified in Figure 5H,I; Figure S7B,C, Supporting Information, silencing N‐WASP dramatically abolished the co‐localization of VAPA with the ARP2/3 complex, consequently resulting in a reduction of the VAPA‐enriched LOs‐induced osteoclast fusion and TRAP enzymatic activity. Taken together, these results demonstrated that N‐WASP is a critical factor for the VAPA‐enriched LOs‐induced actin cytoskeletal remodeling and osteoclastogenesis.

### 2.11 VAPA Activates N‐WASP Via Dual Mechanisms

**Figure 6:**
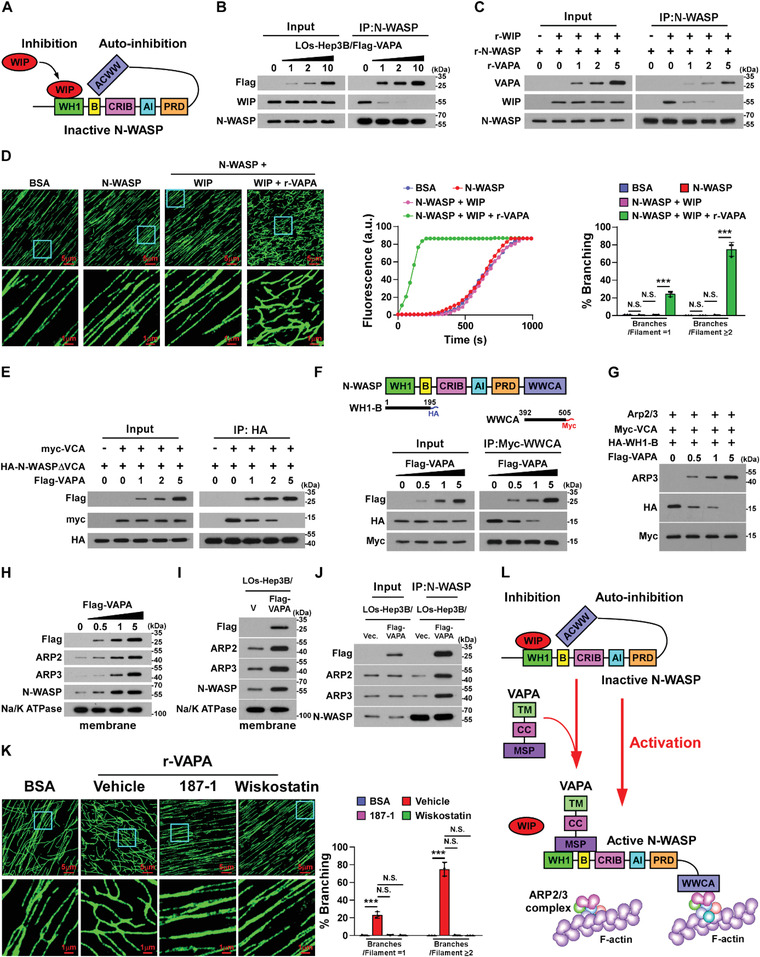
VAPA activates N‐WASP via dual mechanisms. A) Model of WIP‐mediated inhibition and intramolecular auto‐inhibition of N‐WASP. B) Co‐IP assays using anti‐N‐WASP antibody and IB analysis of expression of Flag tagged‐VAPA, WIP, and N‐WASP in the membrane of pre‐osteoclasts treated with the indicated LOs derived from Hep3B/Flag‐VAPA cells. Hep3B cells were transfected with 0, 1, 2, and 10 µg Flag‐VAPA plasmids. C) In vitro co‐IP assays using anti‐N‐WASP antibody in the indicated system and IB analysis of expression of VAPA, WIP, and N‐WASP. D) Left: IF images of polymerized Alexa488‐actin in the indicated polymerization system. Scale bar, 5 µm or 1 µm. Middle: quantification of fluorescence of Alexa488‐actin polymerization in the left panel. Right: quantification of percent branching subcategorized into the percent of total filaments with one branch and the percent of total filaments with two or more branches. E) Co‐IP assays using anti‐HA antibody in the 293 T cells transfected with myc tagged‐VCA, HA tagged‐N‐WASP∆VCA, and 0, 1, 2, and 5 µg Flag tagged‐VAPA plasmids and IB analysis of the levels of the indicated proteins. F) Co‐IP assays using anti‐myc antibody in the 293 T cells transfected with HA tagged‐WH1‐B and Myc tagged‐WWCA and 0, 0.5, 1, and 5 µg Flag tagged‐VAPA plasmids and IB analysis of the levels of the indicated proteins. G) IB analysis of the levels of ARP3, HA, and Myc in the membrane of 293 T cells transfected with ARP2/3, myc tagged‐VCA, HA tagged‐WH1‐B, and 0, 0.5, 1, and 5 µg Flag tagged‐VAPA plasmids. H) IB analysis of expression of Flag tagged‐VAPA, ARP2, ARP3, and N‐WASP in the membrane of 293 T cells transfected with 0, 0.5, 1, and 5 µg Flag tagged‐VAPA plasmids. Na/K ATPsae served as the loading control. I) IB analysis of the levels of Flag tagged‐VAPA, ARP2, ARP3, and N‐WASP in the membrane of pre‐osteoclasts treated with LOs derived from Hep3B/vector or Hep3B/Flag‐VAPA cells. Na/K ATPase served as the loading control. J) Co‐IP assays using anti‐N‐WASP antibody and IB analysis of the levels of Flag‐VAPA, ARP2, ARP3, and N‐WASP in the membrane of pre‐osteoclasts treated with LOs derived from Hep3B/vector or Hep3B/Flag‐VAPA cells. K) Left: IF images of polymerized Alexa488‐actin in the polymerization system treated with BSA or recombinant VAPA protein with vehicle, or 187‐1, or wiskostatin. Scale bar, 5 µm or 1 µm. Right: quantification of the percent branching subcategorized into the percent of total filaments with one branch and the percent of total filaments with two or more branches. L) Model of LOs‐loaded VAPA activates N‐WASP via disruption WIP‐mediated inhibition and intramolecular auto‐inhibition of N‐WASP. Each error bar represents the mean ± SD of three independent experiments. Significant differences were determined by one‐way ANOVA with Tukey's multiple comparison test (D,K). *** *P* < 0.001 and N.S. > 0.05.

The N‐WASP activity could be regulated via multiple mechanisms, such as intramolecular auto‐inhibition via interaction of the WWCA domain with the B domain of N‐WASP and WASP‐interacting protein (WIP)‐mediated inhibition through interaction with the WH1 domain of N‐WASP^[^ ^19^ ^]^ (**Figure** **6**A). Our abovementioned results have demonstrated that VAPA binds to both the WH1 and B domains of N‐WASP (Figure 5F). Therefore, we then examined whether LOs‐loaded VAPA abrogated both WIP‐mediated inhibition and N‐WASP auto‐inhibition. Co‐IP assays revealed that treatment with VAPA‐enriched Los, derived from Hep3B/Flag‐VAPA cells, competitively reduced the WIP/N‐WASP interaction in osteoclasts in a dose dependent manner (Figure 6B). This competitive effect of VAPA on the WIP/N‐WASP interaction was further confirmed using in vitro pull‐down assays (Figure 6C). Consistently, in vitro actin nucleation assays revealed that the addition of VAPA protein drastically relieved the inhibitory effect of WIP on N‐WASP‐mediated actin polymerization and branched actin filaments formation (Figure 6D). Taken together, these results indicate that VAPA activates N‐WASP by abrogating WIP‐mediated inhibition.

We then examined the disruptive effect of VAPA on N‐WASP intramolecular auto‐inhibition. As exemplified in Figure 6E–G, co‐IP assays indicated that overexpressing VAPA drastically decreased the interaction of the VCA fragment with the WH1 fragment or with the N‐WASP∆VCA‐mutant in a dose dependent manner, which resulted in increased association of the VCA with ARP3. These results suggests that VAPA disrupts N‐WASP intramolecular auto‐inhibition. Consistent with the previous reports that inactive N‐WASP exists in the cytoplasm and only activated N‐WASP could induce actin assembly on the cytosolic surface of cellular membranes,^[^ ^19^, ^20^ ^]^ we found that either overexpressing VAPA or treatment with the VAPA‐enriched LOs led to obvious elevation of the N‐WASP level, accompanied with increased N‐WASP‐associated ARP2/3 complex, in the membrane of osteoclasts (Figure 6H–J). Moreover, in vitro actin nucleation assays showed that treatment with either 187‐1 or Wiskostatin, two inhibitors that stabilize the auto‐inhibited conformation of N‐WASP, significantly inhibited the promotive effect of VAPA on actin assembly (Figure 6K; Figure S8A, Supporting Information), which provided further evidence that VAPA is involved in disrupting the intramolecular auto‐inhibition of N‐WASP. Taken together, these results indicate that LOs‐loaded VAPA activates N‐WASP via dual mechanisms (Figure 6L).

### 2.12 VAPA‐Enriched LOs Induce Osteoclastogenesis Analyzed in Bone Organoid Model

**Figure 7:**
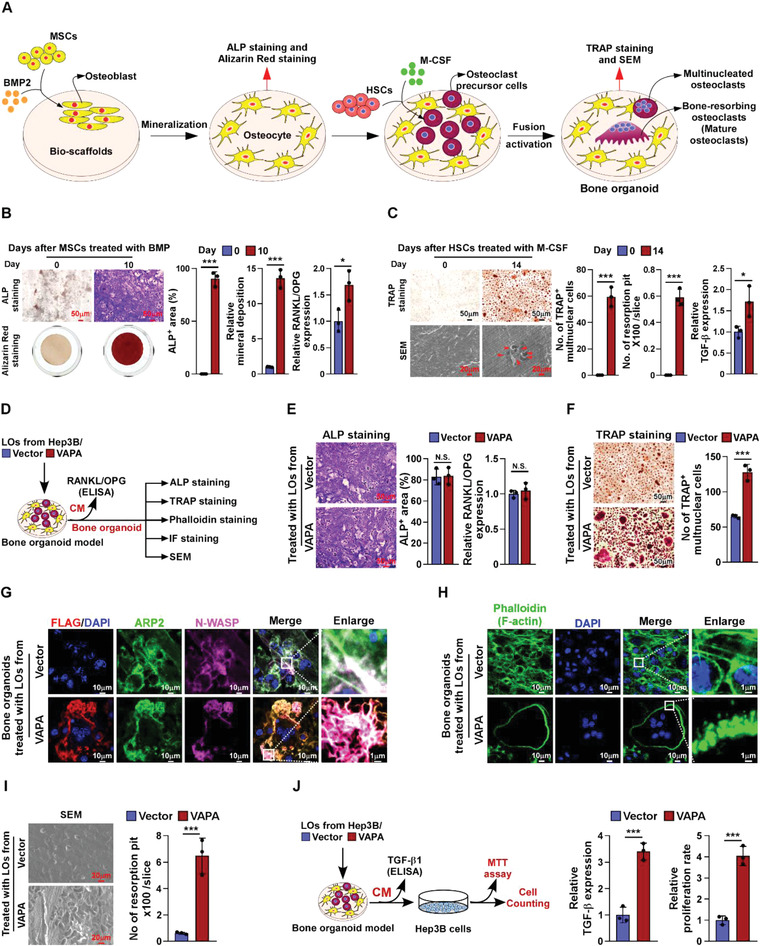
LOs‐loaded VAPA promotes osteoclastogenesis analyzed in bone organoid model. A) Experimental procedure to establish a bone organoid model. B) Left: Representative image of ALP staining and alizarin red mineral staining of osteoblasts derived from MSCs treated with BMP on days 0 and 10. Scale bar, 50 µm. Right: Quantification of percentage of ALP^+^ area and relative mineral deposition from experiment in the left panel, and ELISA analysis of RANKL/OPG ratio in conditional medium (CM) from BMP‐treated bone organoid model on days 0 and 10. C) Left: Representative image of TRAP staining of osteoclasts derived from HSCs and bone resorption pits from bone organoid model treated with M‐CSF on days 0 and 14. Scale bar, 50 µm and 20 µm. Right: Quantification of number of TRAP^+^ multinuclear cells, resorption pits per slice and TGF‐*β* level from experiment in the left panel. D) Schematic illustration of LOs‐induced osteoclastogenesis and bone metastasis using bone organoid model analyzed by ELISA, ALP staining, TRAP staining, phalloidin staining, IF staining, and SEM scanning assays. E) Left: Representative image of ALP staining of osteoblasts in the indicated LOs‐treated bone organoid model. Scale bar, 50 µm. Right: Quantification of percentage of ALP^+^ area from experiment in the middle panel and ELISA analysis of the RANKL/OPG ratio in CM from the indicated LOs‐treated bone organoid model. F) Left: Representative image of TRAP staining in the indicated LOs‐treated bone organoid model. Scale bar, 50 µm. Right: Quantification of number of TRAP^+^ multinuclear cells. G) Representative IF staining images using anti‐Flag, anti‐ARP2, and anti‐N‐WASP antibody in the indicated LOs‐treated bone organoid model. Scale bar, 10 and 1 µm. H) Representative IF staining image of phalloidin (F‐actin) in the indicated LOs‐treated bone organoid model. Scale Bar, 10 and 1 µm. I) Representative image of scanning electron microscopy (SEM) (left) and quantification of the number of resorption pits per slice (right) in the indicated LOs‐treated bone organoid model. Scale bar, 20 µm. J) Left: schematic illustration of LOs‐induced “vicious cycle” between cancer cells and bone organoids. Middle: ELISA analysis of TGF‐*β*1 levels in the CM derived from the indicated LOs‐treated bone organoid model. Right: MTT assay analysis of proliferation rate of Hep3B cells from experiment in left panel. Each error bar represents the mean ± SD of three independent experiments. Significant differences were determined by one‐way ANOVA with Tukey's multiple comparison test (B,C,E,F,I,J). * *P* < 0.05, *** *P* < 0.001, and N.S. > 0.05.

It has been reported that the bone organoid could provide a highly physiologically relevant bone microenvironment, which reproduces bone tissue complexity and bone remodeling processes.^[^ ^21^ ^]^ Therefore, we established the bone organoid model as previous reports using the section of demineralized bovine compact bone to mimic the dense structural collagen matrix of the unmineralized osteoid, and mesenchymal stem cells (MSCs), treated with the bone morphogenic protein (BMP) to differentiate into the osteoblast/osteocyte population (**Figure** **7**A,B), and hematopoietic stem cells (HSCs), treated with macrophage colony‐stimulating factor (M‐CSF) to differentiate into osteoclast precursor cell^[^ ^21^, ^22^ ^]^ (Figure 7A,C). Then, the bone organoid model was employed to examine the effect of VAPA‐enriched LOs on osteoclastic pre‐metastatic niche formation and HCC bone metastasis (Figure 7D–J). Consistent with the in vivo and in vitro results (Figure 2; Figures S3 and S4, Supporting Information), the bone organoid model showed that treatment with Hep3B/VAPA‐derived LOs had no effect on osteoblast differentiation, as indicated by unaltered ALP^+^‐osteoblasts/osteocyte and the RANKL/OPG ratio (Figure 7E) but significantly increased the number of TRAP^+^‐multinuclear osteoclasts (Figure 7F) compared with Hep3B/Vector‐derived LOs treatment. These results provided further evidence that VAPA‐enriched LOs contributed to osteoclastogenesis. Moreover, immunofluorescent staining assays revealed that LOs‐loaded VAPA was co‐localized with ARP2 and N‐WASP in osteoclasts cultured on the bone organoid model and significantly promoted the cytoskeletal organization to form podosomes of osteoclasts (Figure 7G,H). Importantly, the surface of the slice in bone organoid model was severely eroded by treatment with LOs from VAPA‐overexpressing Hep3B cells, as indicated by the increased formation of resorption pits analyzed via scanning electron microscopy (SEM) (Figure 7I). Accompanying severely eroded slice, treatment with VAPA‐overexpressing Hep3B cells‐derived LOs significantly elevated the bone organoid model‐released TGF‐*β*, consequently resulting in higher growth rate of Hep3B cells (Figure 7J). Taken together, these results further support the notion that VAPA‐enriched LOs plays crucial role in osteoclastic pre‐metastatic niche formation and HCC bone metastasis.

### 2.13 Blocking N‐WASP Prevents VAPA‐Enriched LOs‐Induced HCC Bone‐Metastasis

**Figure 8:**
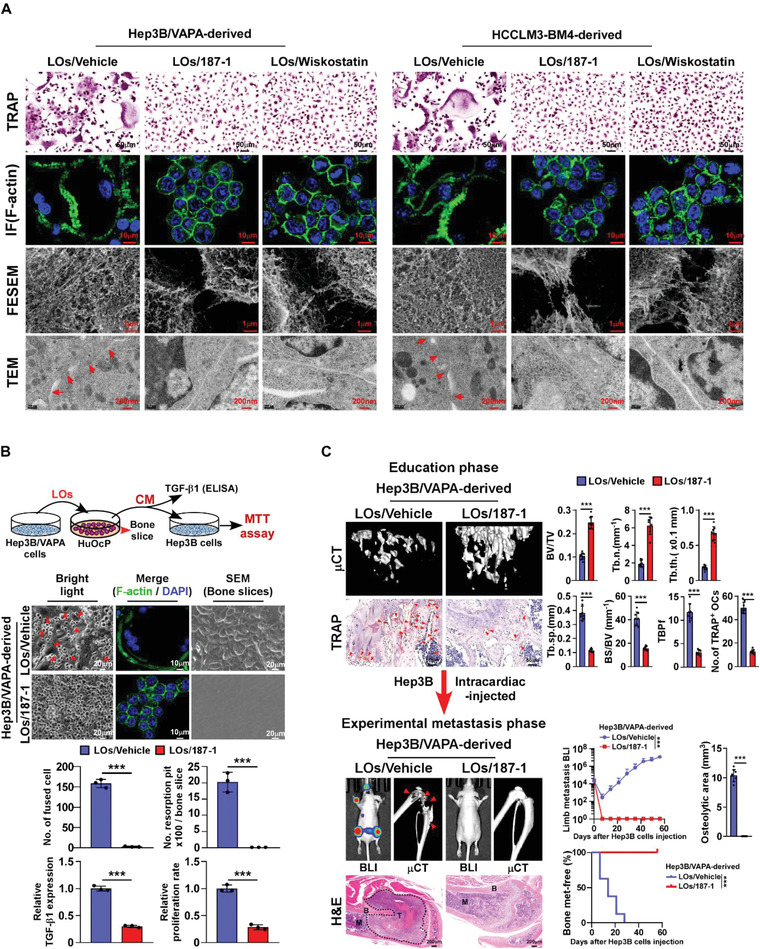
LOs‐packed N‐WASP inhibitor inhibits LOs‐induced osteoclastogenesis and bone metastasis. A) Representative images of TRAP staining, F‐actin staining, and FESEM and TEM in the pre‐osteoclasts treated with LOs derived from vehicle‐ or 187‐1‐, or wiskostatin‐treated Hep3B/VAPA cells (left) and HCCLM3‐BM4 cells (right). Scale bar, 50 µm or 10 µm or 1 µm or 200 nm. B) Upper: schematic illustration of LOs‐induced “vicious cycle” between cancer cells and osteoclasts. Middle: phase contrast and F‐actin staining images of pre‐osteoclasts derived from vehicle‐ or 187‐1‐treated Hep3B/VAPA cells and the indicated SEM images of bone slice. Lower: quantification of the number of fused multinuclear cells and resorption fit, TGF‐*β*1 levels, and proliferation rate of Hep3B cells from the experiment in upper panel. C) Upper‐left: µCT images of bone trabecular section and TRAP histological images of TRAP^+^‐osteoclasts along the bone interface from representative mice treated with LOs derived from vehicle‐ or 187‐1‐treated Hep3B/VAPA cells for education phase (*n* = 8 per group). Scale bar, 50 µm. Upper‐right: quantification of bone parameters and TRAP^+^‐osteoclasts from representative mice in education phase (*n* = 8 per group). Lower‐left: BLI and µCT, and histological (H&E) images of bone tumor and lesions from representative mice in experimental metastasis phase (*n* = 8 per group). Scale bar, 200 µm. Lower‐right: normalized BLI signals of bone metastases, quantification of the µCT osteolytic lesion area, and Kaplan–Meier bone metastasis‐free survival curve of mice in experimental metastasis phase (*n* = 8 per group). Each error bar represents the mean ± SD of three independent experiments. Significant differences were determined by one‐way ANOVA with Tukey's multiple comparison test (B,C). *** *P* < 0.001.

We then examined the effect of inhibiting N‐WASP activity on VAPA‐enriched LOs‐induced osteoclastogenesis. Recently, it was demonstrated that EVs could be used as therapeutic drug carriers to deliver diversified anti‐cancer molecules, such as small interfering RNAs (siRNAs) or pharmacologically active compounds.^[^ ^23^ ^]^ We next incorporated N‐WASP inhibitors 187‐1 or Wiskostatin into VAPA‐enriched LOs derived from VAPA‐overexpressing Hep3B cells or HCCLM3‐BM4 cells. TEM analysis showed that incorporation of 187‐1 or Wiskostatin has no impact on the morphology and size of VAPA‐enriched LOs. Prominently, treatment with either LOs/187‐1 or LOs/Wiskostatin significantly inhibited the inductive effects of VAPA‐enriched LOs on the BAN formation in pre‐osteoclasts, the actin filament density, and the ZLS between fused pre‐osteoclasts as well as the numbers of TRAP^+^‐multinuclear osteoclasts and the TRAP enzymatic activity (**Figure** **8**A; Figure S9A, Supporting Information). Meanwhile, the VAPA‐enriched LOs‐induced formation of resorption pits on bone slice, the bone slice‐released TGF‐*β*, and the relative growth rate of HCC cells decreased significantly in response to LOs/187‐1 or LOs/Wiskostatin treatment (Figure 8B). These results further support the notion that VAPA‐enriched Los induced osteoclastogenesis via activation of N‐WASP‐mediated actin cytoskeleton remodeling.

Furthermore, the in vivo effect of blocking N‐WASP activity on HCC‐BM was examined. Mice were educated with either LOs/vehicle or LOs/187‐1 for 14 days. As exemplified in Figure 8C, LOs/187‐1 treatment dramatically inhibited the educated effect of VAPA‐enriched LOs, which displayed smaller osteolytic lesions, less alteration of bone microarchitecture, and decreased numbers of TRAP^+^‐osteoclasts in the bone surface area. These results suggested that blocking N‐WASP activity could inhibit bone pre‐metastatic niche formation in vivo. Strikingly, further in vivo experimental metastatic assays showed that the promotive effect of VAPA‐enriched LOs education on the HCC bone‐metastasis was also prevented by LOs/187‐1 treatment, as indicated by non‐detectable bone metastatic lesions in the LOs/187‐1‐treated mice, which resulted in longer bone‐metastasis‐free survival (Figure 8C). Therefore, our results demonstrate that blocking N‐WASP activity prevents VAPA‐enriched LOs‐induced bone pre‐metastatic niche formation and HCC bone‐metastasis.

## 3 Discussion

Distant specific‐organ metastasis, termed as metastatic organo‐tropism, is one of the most catastrophic hallmarks of malignant tumors and accounts for most cancer‐related mortalities. However, the molecular mechanisms underlying organ‐specific metastasis are poorly understood. Recently, increasing evidence has demonstrated that EVs function as crucial mediators of bi‐directional tumor–host cell interactions that contribute to metastasis and also especially organ‐specific metastasis. Tumor cells share EVs with the cells in distant organ, and then, alter their gene expression patterns to establish a favorable microenvironment, described as the pre‐metastatic niche, which promotes tumor cells survival and growth.^[^ ^24^ ^]^ In particular, growing evidences support the view that tumor‐derived EVs are involved in the communication between tumor cells and bone cells in the bone‐metastatic niche before bone metastasis.^[^ ^25^ ^]^ In the current study, we found that HCC‐derived LOs, atypically large (1–10 µm diameter) cancer‐derived EVs originating from membrane blebs, exhibited significant potential to induce bone pre‐metastatic niche formation by promoting osteoclastogenesis, consequently resulting in HCC bone‐specific metastasis. Mechanically, LOs‐delivered VAPA protein directly interacts with and activates N‐WASP to promote the ARP2/3‐meidated actin nucleation, leading to cytoskeleton remodeling‐derived osteoclasts fusion and activation. Therefore, our results revealed a plausible mechanism underlying bone‐tropism of HCC.

It has been well‐demonstrated that tumor cell‐induced osteoclastogenesis is a prerequisite for bone pre‐metastatic niche formation. For instance, it has been reported that HCC‐secreted LGALS3‐induced pre‐metastatic niche was through promoting osteoclast fusion and podosome formation, consequently resulting in HCC bone metastasis.^[^ ^8c^ ^]^ Zhao et al. found that long noncoding RNA H19‐mediated HCC bone‐metastasis was through induction of osteoclastogenesis via suppression of osteoprotegerin (OPG).^[^ ^10^ ^]^ Osteoclastogenesis is a multi‐complex procedure that includes many stages, including differentiation to mono‐nuclear pre‐osteoclasts, fusion to form multi‐nuclear mature osteoclasts, and activation to form bone resorbing osteoclasts.^[^ ^26^ ^]^ Actin cytoskeletal reorganization to form the BAN, ZLS, and podosome has been reported to be critical for all these steps.^[^ ^17^, ^26^, ^27^ ^]^ N‐WASP‐mediated ARP2/3 complex activation plays a crucial role in the reorganization of the actin cytoskeleton via modulation of actin filament nucleation and branching.^[^ ^28^ ^]^ However, the N‐WASP activity is commonly inhibited through auto‐inhibition via intramolecular interaction of the WWCA domain with the B domain of N‐WASP and by WIP‐mediated inhibition through interaction with the WH1 domain of N‐WASP.^[^ ^19a^ ^]^ Meanwhile, multiple intracellular molecules, including phosphatidylinositol 4,5‐bisphosphate (PIP2) and cell division cycle 42 (CDC42), were reported to be involved in N‐WASP activation, which consequently activates the ARP2/3 complex in a spatially and temporally appropriate manner. Interestingly, we found that the VAPA cargoed by HCC‐derived LOs significantly activated N‐WASP/ARP2/3 signaling but had no impact on Cdc42 activity and PIP2 level in osteoclast cells. Furthermore, our results demonstrated that VAPA directly interacted with and activated N‐WASP via disruption of its intramolecular auto‐inhibition and WIP‐mediated inhibition, which consequently resulted in osteoclast actin cytoskeletal remodeling and osteoclastogenesis. Therefore, our results unveil a plausible role of VAPA in remodeling of the osteoclast actin cytoskeleton and provide a new mechanism underlying N‐WASP activation in osteoclasts.

Over the past few decades, the overall survival of patients with cancer, including HCC, has been considerably improved because of the marked progress in approaches to early diagnosis, surveillance, prevention, and multidisciplinary treatments. However, this consequently offers the extra time for development of metastatic foci at distant organs. Bone is the second most frequent site of metastasis for HCC, with a metastasis rate of ≈16.1–38.5% among the patients with extrahepatic disease.^[^ ^29^ ^]^ Bone metastasis (BM) also occurs in 11.7% of HCC patients who undergo curative resections.^[^ ^30^ ^]^ The consequences of BM are often devastating, which the median survival time of HCC with bone metastasis (HCC‐BM) is only 5.0 months.^[^ ^31^ ^]^ Clinically, bone metastasis of hepatocellular carcinoma (HCC) is typically presented as osteolytic bone metastasis, which is characterized by increased osteoclast‐mediated abnormal bone destruction.^[^ ^32^ ^]^ Although employment of 18F‐fluorodeoxyglucose positron emission computerized tomography (FDG‐PET/CT) or bone scintigraphy (BS) significantly improves the detection rate of loco‐regional BM, it is usually incurable once tumors metastasize to bone. Therefore, there is still an urgent need to identify clinical biomarkers to select HCC patients at high risk of BM. Herein, we found that prior to bone metastasis, VAPA‐enriched LOs derived from bone‐metastatic HCC cells could induce drastic changes in the bone microarchitecture, suggesting that LOs‐cargoed protein plays crucial roles in bone pre‐metastatic niche formation. Importantly, ELISA analysis showed that the serum level of VAPA was significantly higher in the mice bearing bone‐metastatic HCC cells but was not detectable in the mice bearing non‐metastatic HCC cells. Meanwhile, the serum level of VAPA, which was at a very low level in healthy serum, was significantly higher in HCC‐BM patients than that in HCC patients without bone‐metastasis. Therefore, our results suggest that the serum VAPA level might be a potential clinical diagnostic biomarker to predict HCC‐BM. Considering that bone metastasis is a common complication for many types of cancer, further investigation of whether the serum VAPA level correlates with BM from other cancer is warranted.

Currently, there is no adequate curative regimen for patients with BM. This is despite the fact that bisphosphonates, the potent inhibitors of osteoclast‐mediated bone resorption, and denosumab, a humanized monoclonal antibody against RANKL, have been approved by the FDA to treat and prevent metastatic bone disease. However, because of the severe side effects of prolonged bisphosphonate or denosumab treatment, such as atypical bone fractures and jaw bone problems, international practice guidelines have not stated a preference for either denosumab or bisphosphonates.^[^ ^33^ ^]^ Therefore, novel strategies to prevent and treat early‐stage bone disease are urgently required. Due to the critical role in metastatic organotropism, recent studies have demonstrated that EVs and their components could be considered as targets for therapeutic intervention via reducing the load of circulating EVs or blocking their crucial components of extracellular vesicles. For instance, it has been reported that the exosomal integrins *α*6*β*4 and *α*6*β*1 are associated with lung metastasis, while exosomal integrin *α*v*β*5 was linked to liver metastasis and targeting the integrins *α*6*β*4 and *α*v*β*5 decreased exosome uptake, as well as lung and liver metastasis, respectively.^[^ ^34^ ^]^ Rodrigues et al. reported that silencing CEMIP, which was enriched in brain‐tropic EVs in comparison to lung‐tropic or bone‐tropic EVs, dramatically hindered the brain metastasis of breast cancer cells.^[^ ^35^ ^]^ Consistently, our data demonstrated that silencing VAPA in HCC cells consequently resulted in VAPA reduction in HCC‐derived LOs, or treatment with anti‐VAPA antibody significantly abolished HCC‐derived LOs‐induced formation of the bone‐metastatic niche and HCC bone metastasis. Importantly, utilizing HCC cell‐derived LOs as N‐WASP inhibitor delivery vehicles drastically abolished the inductive effect of VAPA‐enriched LOs on bone‐metastatic niche formation and HCC bone metastasis. Therefore, our results not only provide valuable insights to understand the mechanism and pathophysiology of bone metastatic tropism of HCC but also might represent a potential novel strategy to treat HCC bone metastasis.

## 4 Experimental Section

### Cell Lines and Cell Culture

The HCCLM3 and Hep3B HCC cell lines, human embryonic kidney cells 293FT, and murine Hepa1‐6 hepatoma cell line were purchased from American Type Culture Collection (ATCC, Manassas, VA) and were grown in Dulbecco's modified Eagle's medium (DMEM) (Gibco, Grand Island, NY) supplemented with 10% fetal bovine serum (Gibco, Grand Island, NY). All the cell lines were tested for mycoplasma contamination and were authenticated by short tandem repeat (STR) fingerprinting at Medicine Lab of Forensic Medicine Department of the Sun Yat‐sen University.

### Xenografted Tumor Models

All of the animal procedures were approved by the Sun Yat‐sen University Institutional Animal Care and Use Committee (Approval No. SYSU‐IACUC‐2021‐000741). Before cancer cell injection, mice were educated for 2 weeks by intracardial injections of 100 µL of LOs from indicated cells. At the 15 days, intracardiac injections of luciferase expressing Hep3B cells (1 × 10^5^) or Hep1‐6 cells (5 × 10^5^) were done into nu/nu nude mice (5 weeks old) for bone metastasis assays. Anti‐VAPA antibody (abcam) or anti‐rabbit IgG were administered intraperitoneally twice a week at 1 mg kg^−1^, 2 days after implantation. LOs/187‐1 (10^6^ particles/100 µL per mouse) was injected intraperitoneally twice a week. The formation of bone metastases was observed and assessed weekly in mice injected with D‐luciferin (75 mg kg^−1^) by bioluminescence imaging using the IVIS Spectrum In Vivo Imager. At week 5, the osteolytic lesions in mice were observed using SIEMENS micro‐CT system (Inveon). Bone of mice were harvested and prepared for the following analysis.

### Establishment of Bone Organoid Model

The bone organoid model was established using a modification of the method as previously reported.^[^ ^21^, ^22^ ^]^ In brief, 1.0 × 10^4^ MSCs were seeded on demineralized bone matrix in a 24‐well plate and cultured with differentiation medium for 10 days. The ALP staining and alizarin red mineral staining were, respectively used to characterize differentiation and mineralization by MSCs‐derived osteoblasts in osteogenic differentiation medium on day 10. Next, 1.0 × 10^5^ HSCs were added to each well and induced by 30 ng mL^−1^ M‐CSF (R&D Systems) for 14 days. In addition, TRAP staining and scanning electron microscopy were, respectively used to characterize differentiation and resorption pits were observed by HSCs‐derived osteoclasts after co‐culture for day 14.

### Statistical Analysis

All data were presented as the mean ± standard deviation (SD). *n* represents the number of independent experiments performed on different mice or different batches of cells or different clinical tissues. Statistical analysis was performed using the Student's two‐tailed *t*‐test and one‐way analysis of variance (ANOVA). Bivariate correlations between study variables were calculated by Spearman's rank correlation coefficients. Survival curves were plotted by the Kaplan–Meier method and compared by the log‐rank test. The significance of various variables for survival was analyzed by univariate and multivariate Cox regression analyses. *P* values of 0.05 or less were considered statistically significant. Statistical analysis was performed using the GraphPad Prism 7. Representation of the *p* values was *P < 0.05, **P < 0.01, ***P < 0.001, and N.S.: not significant (*P* > 0.05).

## Conflict of Interest

The authors declare no conflict of interest.

## Author Contributions

Designed the experiments and analyzed data: S.Z. Performed the xenograft tumor experiments: X.L. and S.C. Performed in vitro cell studies: M.L. Performed staining, immunohistochemical and pathological analysis: W.Q. and S.M. Performed the immunoprecipitation and western blot: Y.X. and M.Y. Analyzed mass spectrometry data: X.L. and M.T. Performed the extracellular vesicle isolation and proof experiments: X.W. and Y.H. Performed the actin nucleation assays: Z.L.,R.Y., and A.A. Supervised the whole study and wrote the paper: L.S. and J.L.

## Supporting information

Supporting Information

## Data Availability

The data that support the findings of this study are available from the corresponding author upon reasonable request. The data are not publicly available due to privacy or ethical restrictions.
