# Supplementary material for: Large Oncosome‐Loaded VAPA Promotes Bone‐Tropic Metastasis of Hepatocellular Carcinoma Via Formation of Osteoclastic Pre‐Metastatic Niche
Source: Adv Sci (Weinh). 2022 Sep 28;9(31):2201974. doi: 10.1002/advs.202201974 (PMC9631052; doi:10.1002/advs.202201974)
Supplement: Supplementary file 1 — Supporting Information [file ADVS-9-2201974-s001.pdf]

Large oncosome-loaded VAPA promotes bone-tropic metastasis of hepatocellular carcinoma via formation of osteoclastic pre-metastatic niche

Shuxia Zhang, Xinyi Liao, Suwen Chen, Wanying Qian, Man Li, Yingru Xu, Meisongzhu Yang, Xincheng Li, Shuang Mo, Miaoling Tang, Xingui Wu, Yameng Hu, Ziwen Li, Ruyuan Yu, Ainiwaerjiang Abudourousuli, Libing Song*, Jun Li*

1. Supplementary Figures and Figure Legends…………………………………..…....Page 2-15

2. Supplementary Materials and Methods…………………………………………Page 16-28

3. Supplementary Tables……….……………………………………………….….Page 29-41

Supplementary Figures and Figure Legends

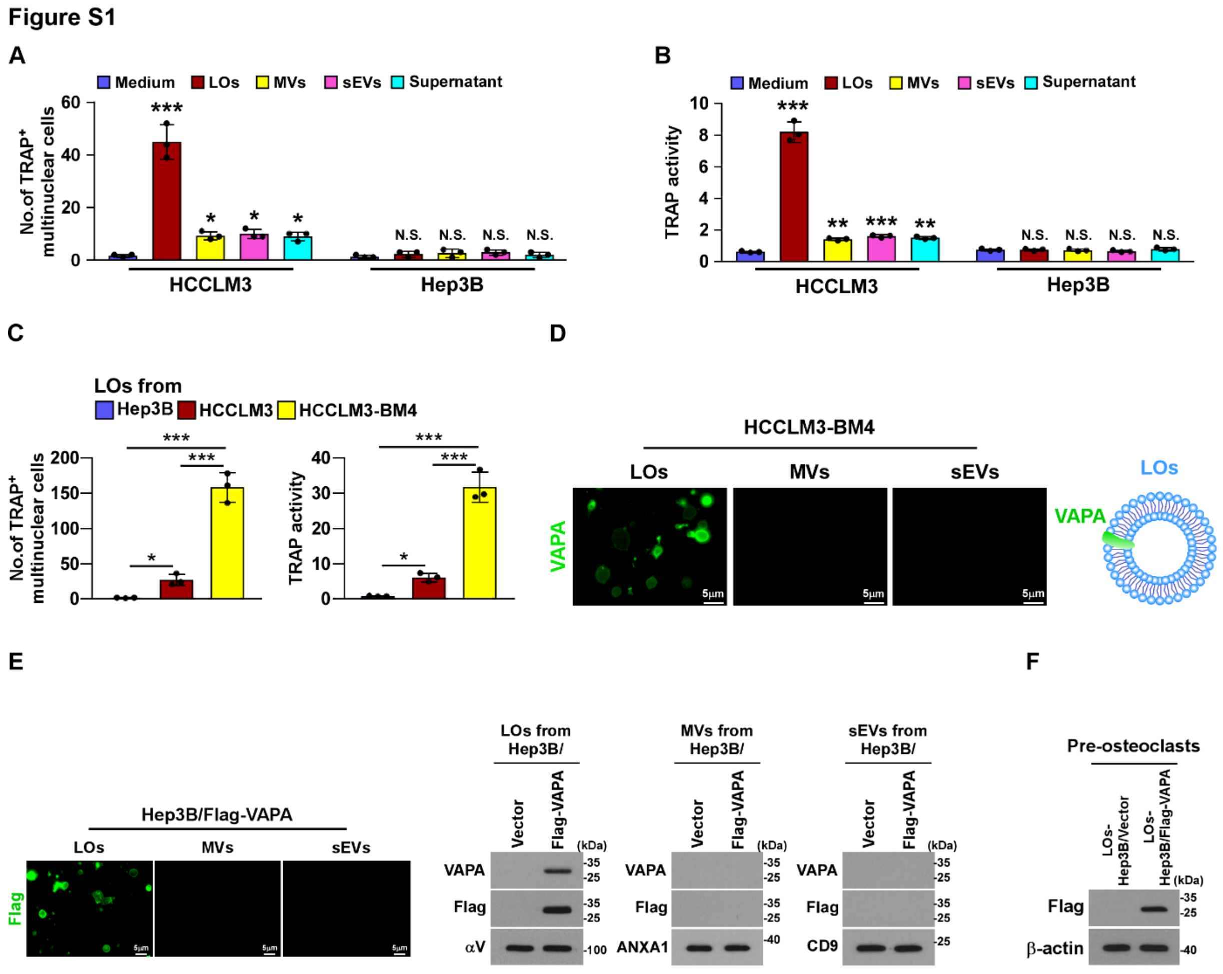

Figure S1. VAPA protein was enriched in LOs derived from bone metastatic HCC cells. (A-B) Quantification of number of TRAP$^+$-multinuclear cells (A) and TRAP activity (B) of pre-osteoclasts treated with the control medium, and LOs, MVs and sEVs derived from HCCLM3 or Hep3B cells. **(C)** Quantification of number of TRAP$^+$-multinuclear cells and TRAP activity of pre-osteoclasts treated with LOs derived from Hep3B, HCCLM3, and HCCLM3-BM4 cells. **(D)** IF staining image of LOs, MVs and sEVs derived from HCCLM3-BM4 cells using Alexa488-anti-VAPA antibody. Scale bar, 5μm. **(E)** Left: IF staining image of LOs, MVs and sEVs derived from Hep3B/VAPA cells using Alexa488-anti-Flag antibody. Scale bar, 5μm. Right: IB analysis of VAPA expression in Hep3B/VAPA cells-derived LOs, MVs and sEVs using anti-VAPA and anti-Flag antibody.

αV-integrin, ANXA1 and CD9 served as the loading control respectively. **(F)** IB analysis of Flag tagged-VAPA in pre-osteoclast treated with LOs derived from Hep3B/Vector and Hep3B/Flag-VAPA cells. β-actin served as the loading control. Each error bar represents the mean ± SD of three independent experiments. Significant differences were determined by One-way ANOVA with Tukey's multiple comparison test (**A-C**). * P < 0.05, *** P < 0.001, N.S. > 0.05.

Figure S2

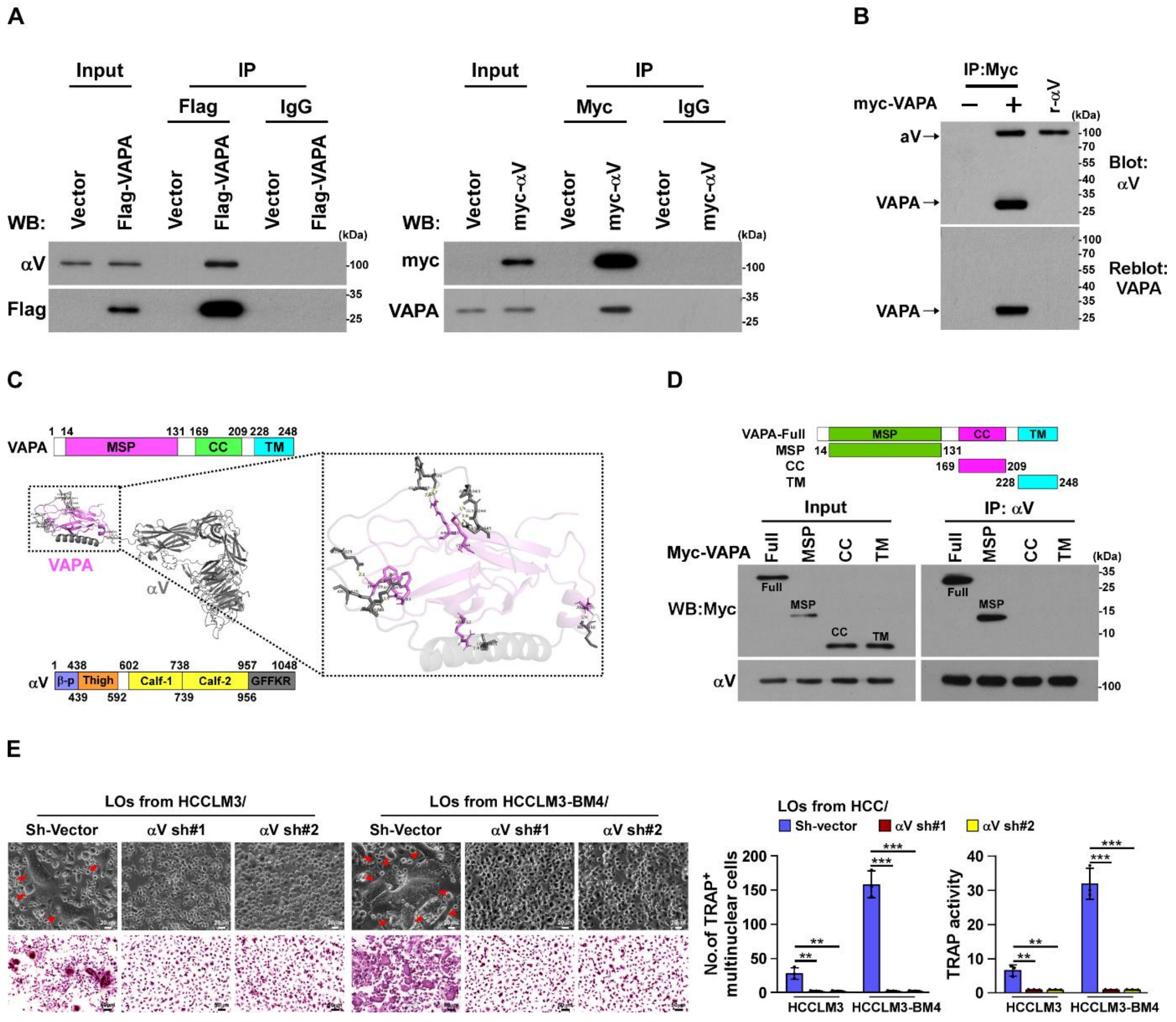

Figure S2. LOs marker αV-integrin interacts with and sorts VAPA into LOs. (A) Co-IP/IB analyses of interaction of VAPA with αV-integrin in the Flag tagged-VAPA transfected HCCLM3-BM4 cells (left) or in the myc-tagged-αV-integrin-transfected HCCLM3-BM4 cells (right). **(B)** Far-western blotting analysis was performed using anti-myc antibody-immunoprecipitated proteins and detected using anti-αV-integrin antibody, and then re-blotted with anti-VAPA antibody. **(C)** Three-dimensional (3D) structure of the interaction region between αV-integrin and VAPA. **(D)** Schematic illustration of the wild-type and truncated VAPA protein (upper) and co-IP assays were performed using anti-αV-integrin antibody in the indicated cells (lower). **(E)** Left: TRAP staining images of pre-osteoclasts treated with LOs derived from the indicated cells. Scale bar, 20μm or 50μm.

Right: quantification of number of $TRAP^+$ multinuclear cells and TRAP activity from experiment in the left panel. Each error bar represents the mean ± SD of three independent experiments. Significant differences were determined by One-way ANOVA with Tukey's multiple comparison test (**E**). ** $P < 0.01$, *** $P < 0.001$.

Figure S3

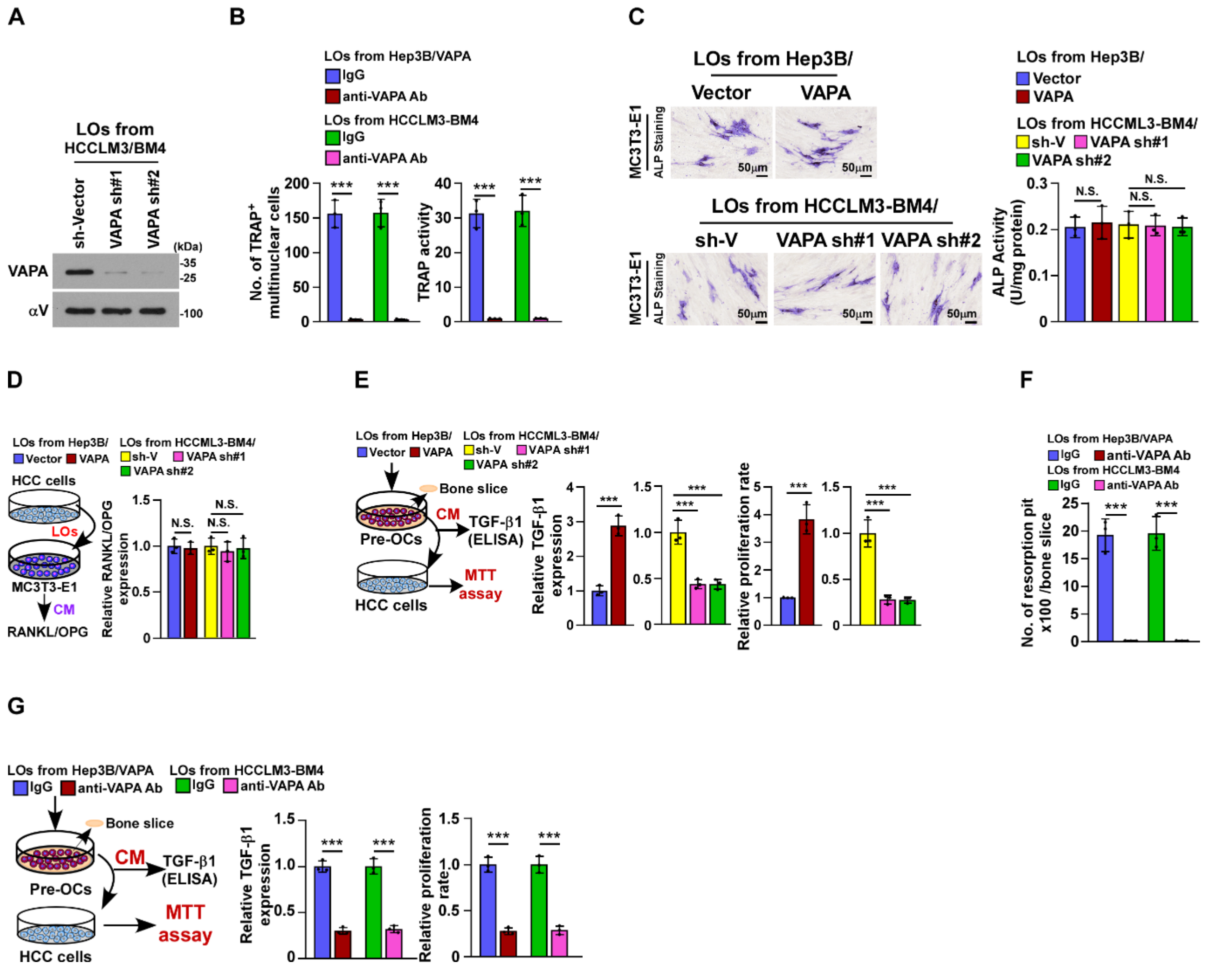

Figure S3. LOs-loaded VAPA promotes osteoclastogenesis and the vicious cycle. (A) IB analysis of VAPA levels in LOs derived from control and VAPA-silenced HCCLM3-BM4 cells. αV-integrin served as the loading control. **(B)** Quantification of number of TRAP$^+$ multinuclear cells and TRAP activity of pre-osteoclasts treated with the indicated cells-derived LOs and IgG or anti-VAPA antibody. **(C)** Left: IF staining image of MC3T3-E1 osteoblasts treated with LOs derived from Hep3B/Vector and Hep3B/Flag-VAPA cells using anti-ALP antibody. Right: Quantification of ALP activity from the experiment in the left panel. **(D)** ELISA analysis of RANKL/OPG ratio in CM from MC3T3-E1 pre-osteoblasts treated with LOs derived from the indicated HCC cells. **(E)** Left: Schematic illustration of LOs-induced "vicious cycle". Middle: ELISA analysis of TGF-β1 level in CM from pre-osteoclasts cultured onto the bone slice in the presence of indicated CM from HCC

cells. Right: MTT assay analysis of proliferation rate of Hep3B cells treated with the indicated CM. **(F)** Quantification of resorption fit in bone slice cultured with pre-osteoclasts treated with the indicated cells-derived LOs and IgG or anti-VAPA antibody. **(G)** Left: schematic illustration of LOs-induced "vicious cycle" between cancer cells and osteoclasts. Middle: ELISA analysis of TGF-β1 levels in CM derived from bone slice co-cultured with pre-osteoclasts under the indicated treatments. Right: MTT assay analysis of proliferation rate of Hep3B cells from experiment in left panel. Each error bar represents the mean ± SD of three independent experiments. Significant differences were determined by One-way ANOVA with Tukey's multiple comparison test (**B-G**). *** $P < 0.001$, N.S. > 0.05.

Figure S4

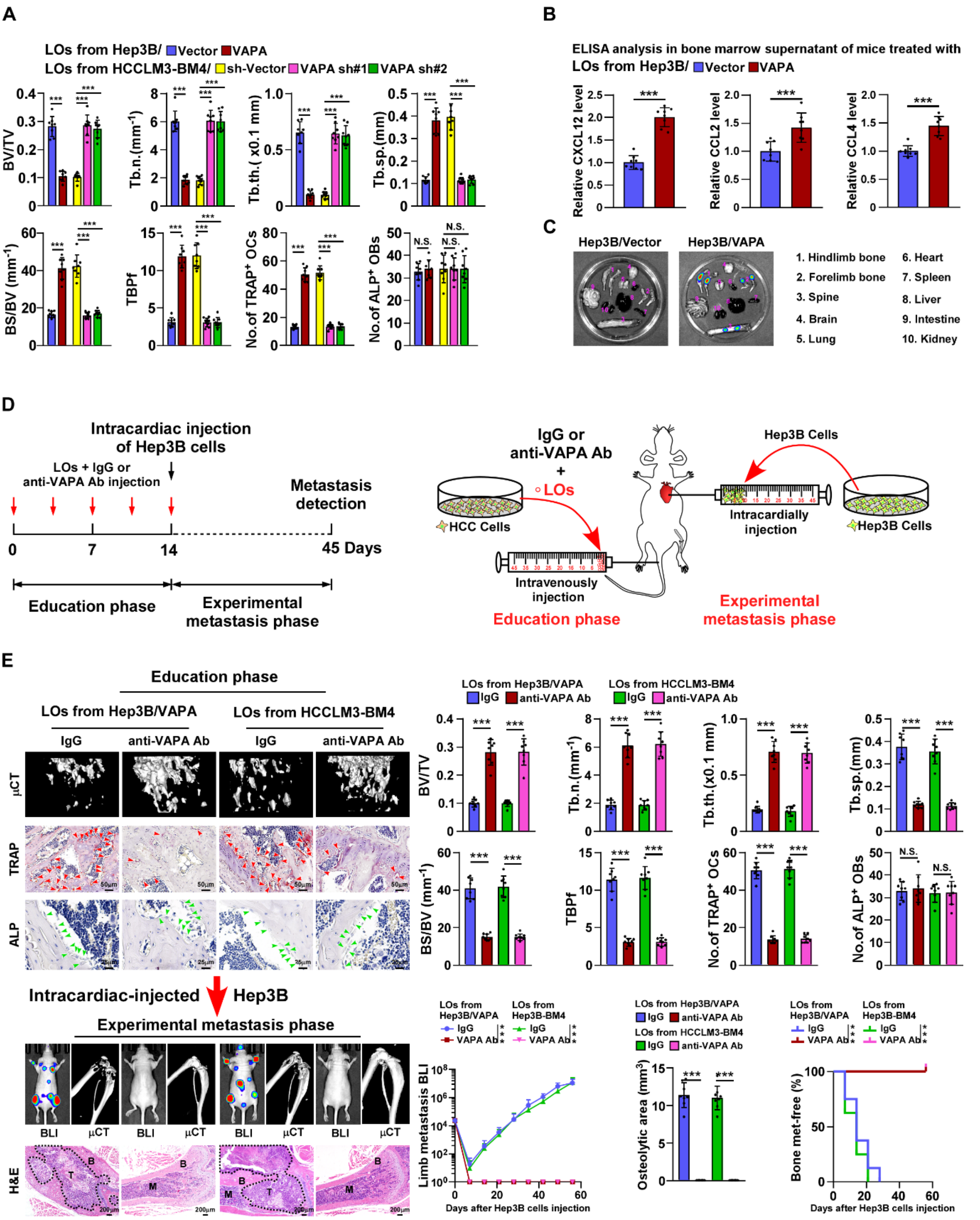

Figure S4. Targeting VAPA inhibits LOs-induced osteoclastogenesis and HCC bone metastasis. **(A)** Quantification of bone parameters and number of TRAP$^+$-osteoclasts/ALP$^+$-osteoblasts from the indicated mice in the education phase of Figure 2D (n = 8/group). **(B)** ELISA analysis of CXCL12, CCL2 and CCL4 level in bone marrow

supernatants of mice treated with indicated LOs from HCC cells. **(C)** BLI images of bone, including hindlimb bone, forelimb bone and spine, but not in brain, lung, heart, spleen, liver, intestine and kidney from representative mice in experimental metastasis phase. **(D)** Schematic illustration of a bone-metastatic mouse model of education phase and experimental metastasis phase *in vivo*. **(E)** Upper-left: μCT images of bone trabecular section (upper), and histological (TRAP and ALP) images (middle and lower) of $TRAP^+$-osteoclasts/ALP^+-osteoblasts along the bone interface from representative mice in education phase (n = 8/group). Scale bar, 50μm or 25μm. Upper-right: quantification of bone parameters and $TRAP^+$-osteoclasts/ALP^+-osteoblasts from representative mice in education phase (n = 8/group). Lower-left: BLI and μCT, and histological (H&E) images of bone tumor and lesions from representative mice in experimental metastasis phase. Scale bar, 200 μm. Lower-right: normalized BLI signals of bone metastases, quantification of the μCT osteolytic lesion area, and Kaplan-Meier bone metastasis-free survival curve of mice from the indicated experimental groups in experimental metastasis phase (n = 8/group). Each error bar represents the mean ± SD of three independent experiments. Significant differences were determined by One-way ANOVA with Tukey's multiple comparison test (**A-B and E**). *** $P < 0.001$, N.S. > 0.05.

Figure S5

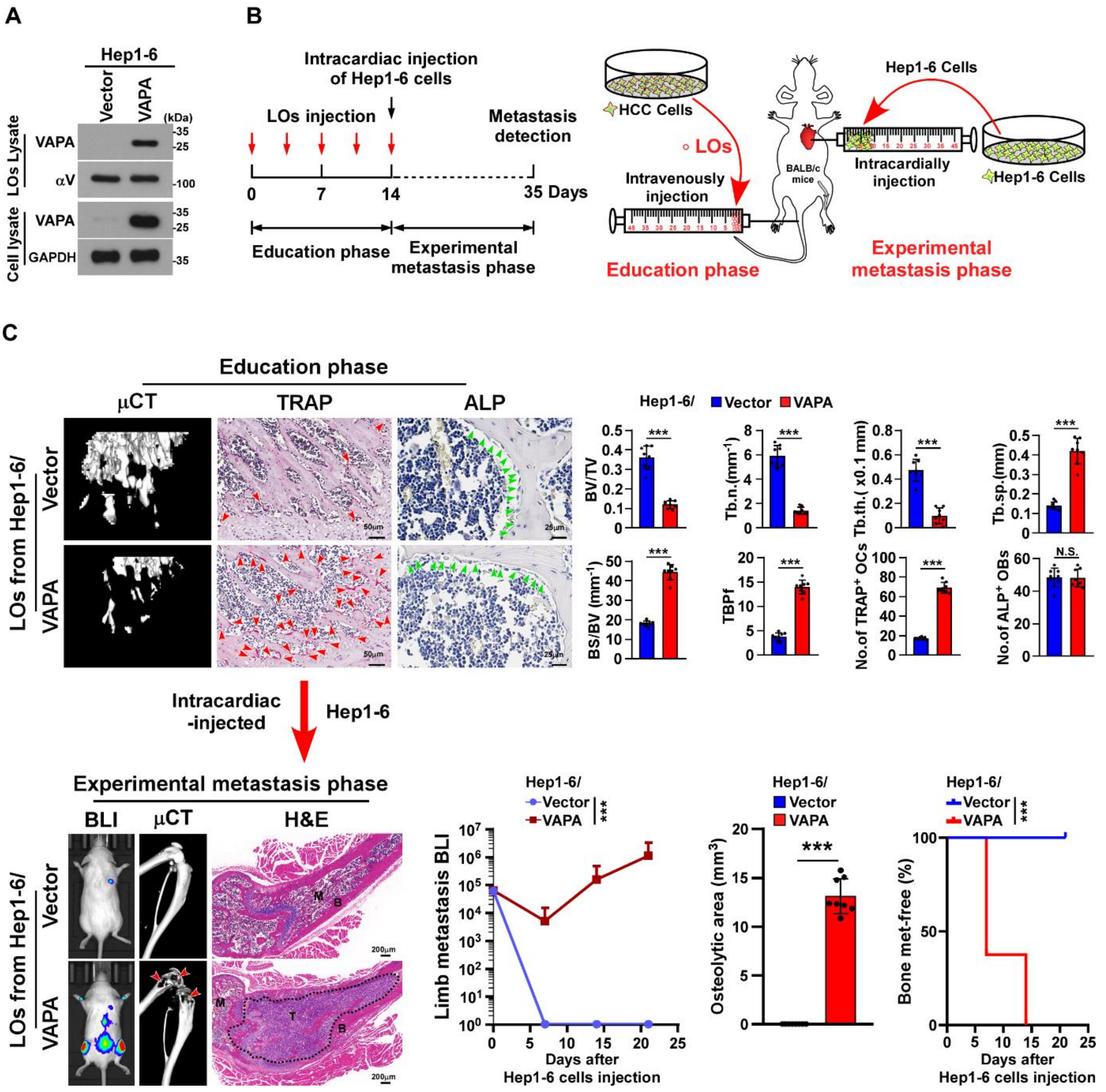

Figure S5. VAPA-loaded LOs induces bone pre-metastatic niche formation and HCC bone metastasis *in vivo*. **(A)** IB analysis of levels of αV-integrin and VAPA in the Hep1-6/Vector or Hep1-6/VAPA cells and LOs. GAPDH served as the loading control. **(B)** Schematic illustration of BALB/c mouse model of the education phase (left) and experimental metastasis phase *in vivo* (right). **(C)** Upper-left: μCT images of bone trabecular section and histological (TRAP and ALP) images of TRAP^{+}-osteoclasts/ALP^{+}-osteoblasts along the bone interface from representative mice treated with LOs derived from Hep1-6/Vector or Hep1-6/VAPA cells for education phase (n = 8/group). Scale bar, 50μm. Upper-right:

quantification of bone parameters, TRAP^{+}-osteoclasts and ALP^{+}-osteoblasts from representative mice in education phase (n = 8/group). Lower-left: BLI and μCT, and histological (H&E) images of bone tumor and lesions from representative mice in experimental metastasis phase (n = 8/group). Scale bar, 200 μm. Lower-right: normalized BLI signals of bone metastases, quantification of the μCT osteolytic lesion area, and Kaplan-Meier bone metastasis-free survival curve of mice in experimental metastasis phase (n = 8/group). Each error bar represents the mean ± SD of three independent experiments. Significant differences were determined by the Student's two-tailed t-test (**C**). *** $P < 0.001$, N.S. > 0.05.

Figure S6

A

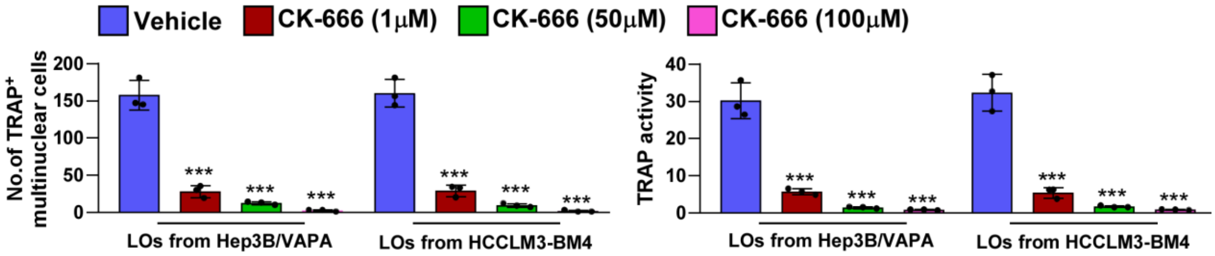

Figure S6. Inhibition of ARP2/3 activity abrogates LOs-induced osteoclastogenesis.

(A) Quantification of number of TRAP$^+$-multinuclear cells (left) and TRAP activity (right) of pre-osteoclasts treated with Hep3B/VAPA- or HCCLM3-BM4 cells-derived LOs plus vehicle or 1μM, 50μM, 100μM CK-666. Each error bar represents the mean ± SD of three independent experiments. Significant differences were determined by One-way ANOVA with Tukey's multiple comparison test (**A**). *** $P < 0.001$.

Figure S7

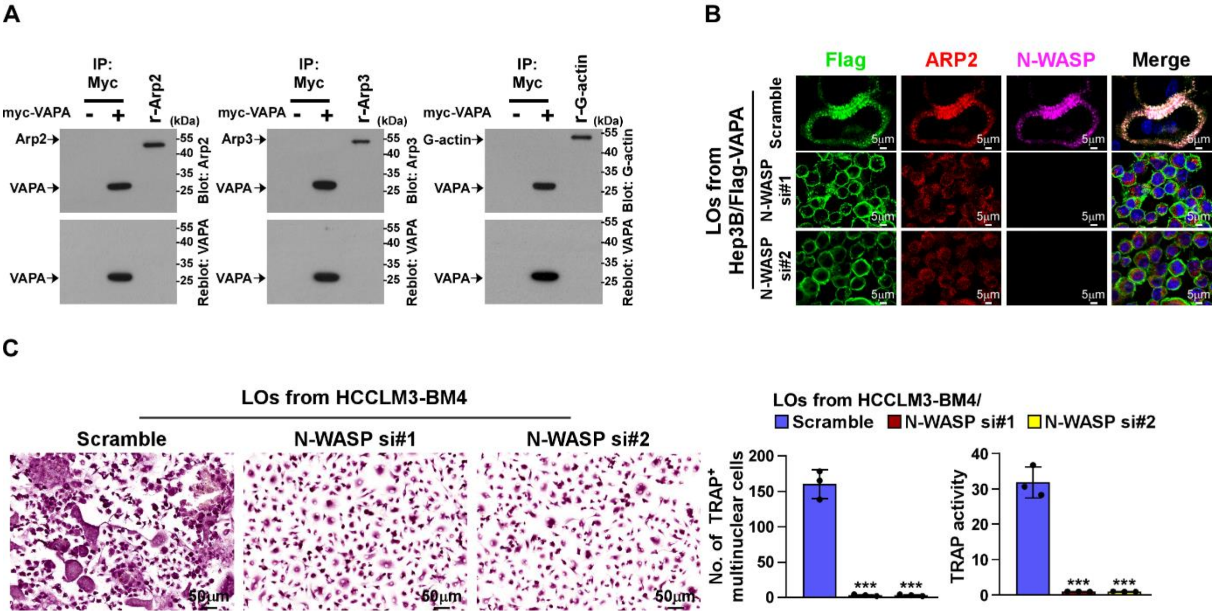

Figure S7. N-WASP is essential for VAPA-induced osteoclastogenesis. (A) Far-western blotting analysis was performed using anti-myc antibody-immunoprecipitated proteins and detected using anti-ARP2 antibody, or anti-ARP3 antibody, or anti-G-actin antibody, and then re-blotted with anti-VAPA antibody. Recombinant ARP2, ARP3 and G-actin served as the control. **(B)** IF staining images of osteoclasts treated with indicated LOs using anti-Flag, anti-ARP2 and anti-N-WASP antibody. Scale bar, 5μm. **(C)** Left: TRAP staining images of N-WASP-silenced pre-osteoclasts treated with the indicated cells-derived LOs. Scale bar, 50μm. Right: quantification of number of $TRAP^+$ multinuclear cells and TRAP activity from experiment in the left panel. Each error bar represents the mean ± SD of three independent experiments. Significant differences were determined by One-way ANOVA with Tukey's multiple comparison test (**C**). *** $P < 0.001$.

Figure S8

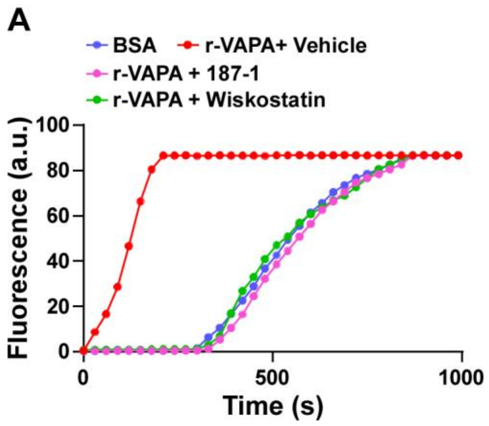

Figure S8. Blocking N-WASP activity inhibits the promotive effect of VAPA on F-actin nucleation. **(A)** Quantification of fluorescence of polymerized Alexa488-actin in polymerization assays as shown in Figure 6K.

Figure S9

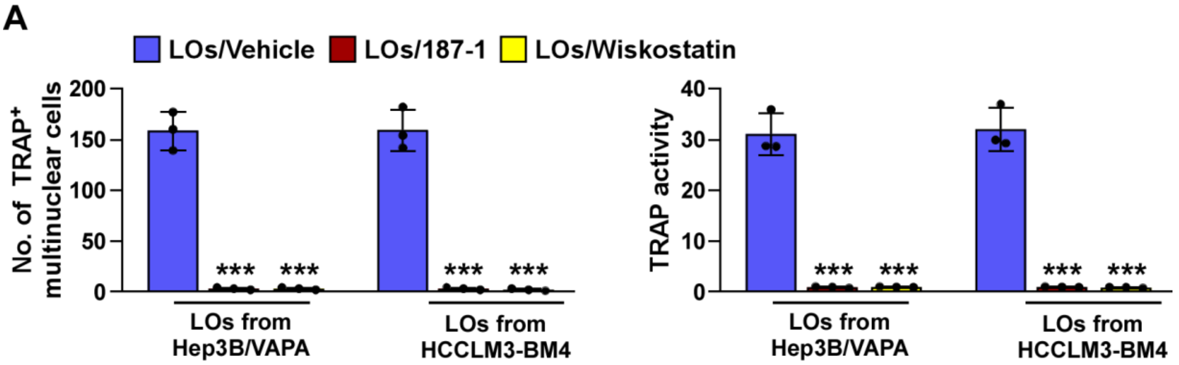

Figure S9. LOs-packed N-WASP inhibitor (LOs/187-1) abrogates the promotive effect of VAPA on osteoclastogenesis. (A) Quantification of number of $TRAP^+$ multinuclear cells (left) and TRAP activity (right) of pre-osteoclasts treated with LOs derived from vehicle, or 187-1, or wiskostatin-treated Hep3B/VAPA or HCCLM3-BM4 cells. Each error bar represents the mean ± SD of three independent experiments. Significant differences were determined by One-way ANOVA with Tukey's multiple comparison test (**A**). *** $P < 0.001$.

Supplementary Materials and Methods

Patient information. This study, which complied with all relevant ethical regulations for work with human participants, was conducted on a total of 21 tumor-adjacent normal liver tissues and 369 paraffin-embedded HCC samples that were histopathologically and clinically diagnosed at the Third Affiliated Hospital and Sun Yat-sen University Cancer Center from 2005 to 2018. Among extrahepatic metastatic HCC samples, 26 primary HCC samples with bone-metastasis and 11 HCC biopsies at bone site, 332 primary HCC samples with other organ metastasis. The study protocols were approved by the Ethical Committee of the Sun Yat-sen University Cancer Center for the use of these clinical materials for research purposes. All Patients' samples were obtained according to the Declaration of Helsinki and each patient signed a written informed consent for all the procedures.

Plasmids, retroviral infection and transfection. The human VAPA, N-WASP, Alpha-V, truncated-VAPA fragments, and truncated-N-WASP fragments were cloned into pCDH vector. ShRNAs targeting VAPA and N-WASP were cloned into the pSuper Retro viral vector. Transfection of short interfering RNAs (siRNAs) or plasmids was performed using the Lipofectamine 3000 reagent (Thermo Fisher Scientific, Waltham, MA, USA) according to the manufacturer's instructions. Stable cell lines expressing VAPA or N-WASP, and N-WASP or VAPA shRNA(s) were generated via retroviral infection and selected for 10 days with 0.5 μg/mL puromycin 48 h after infection.

Chemical reagents. N-WASP inhibitor, 187-1(HY-P1045) was purchased from MedChemExpress (Monmouth Junction, NJ 08852, USA), and Wiskostatin (ab141085) was purchased from Abcam (Cambridge, MA, USA). Arp2/3 complex inhibitor CK-666 (182515) were purchased from Sigma Aldrich (St. Louis, MO, USA).

Co-immunoprecipitation (Co-IP) assay. Cells grown in 100-mm culture dishes were lysed

using 500 μL of lysis buffer [25 mmol/L HEPES (pH 7.4), 150 mmol/L NaCl, 1% NP-40, 1 mmol/L EDTA, 2% glycerol, and 1 mmol/L phenylmethylsulfonyl fluoride (PMSF)]. After being maintained on ice for 30 minutes, the lysates were clarified by microcentrifugation at 12,000 rpm for 10 minutes. To preclear the supernatants, the lysates were incubated with 20 mL of agarose beads (Calbiochem, San Diego, CA, USA) for 1 hour with rotation at 4°C. After centrifugation at 2,000 rpm for 1 minutes, the supernatants were incubated with 20 mL of antibody-cross-linked protein G-agarose beads overnight at 4°C. The agarose beads were then washed six times with wash buffer [25 mmol/L HEPES (pH 7.4), 150 mmol/L NaCl, 0.5% NP-40, 1 mmol/L EDTA, 2% glycerol, 1 mmol/L PMSF]. After removing all the liquid, the pelleted beads were resuspended in 30 mL of 1 M glycine (pH 3), after which, 10 μL 4 × sample buffer was added, the samples were denatured, and the sample components were electrophoretically separated on SDS-PAGE for immunoblot analysis.

Far-Western analysis. Far-Immunoblotting were performed by using the proteins immunoprecipitated by anti-VAPA, anti-N-WASP, anti-ARP2, anti-ARP3, anti-actin and anti-αV-integrin antibodies and human recombinant N-WASP, ARP2, ARP3, G-actin and αV-integrin protein. Briefly, the proteins were separated by SDS-PAGE, and were transferred onto a PVDF membrane. Membranes were then preincubated in 10% skimmed milk for 1 hour at 4°C. As indicated, recombinant α-catenin protein was added at 5μg/mL and incubated at 4°C for 18h. After extensive washing six times with TBST, the membrane was subjected to immunoblotting analysis using indicated antibodies.

Plasma Membrane Protein Extraction. A plasma membrane protein extraction kit (ab65400; Abcam, Cambridge, MA, USA) was used to extract the plasma membrane protein according to the manufacturer's instructions.

Extracellular vesicle isolation from cultured HCC cells. Cells-conditioned culture

medium was collected from approximately 95% confluent HCC cells grown for 72h in 40 ×100mm cell culture dishes with DMEM containing FBS depleted of bovine serum extracellular vesicles (EVs) by 24h ultracentrifugation at 120,000×g, 4°C. The collected media was first subjected to a centrifugation step of 400×g for 10 min at room temperature (RT) to pellet and remove cells. All following centrifugation steps were performed at 4°C. Next, the supernatant was spun at 2,000×g for 20 min to remove debris and apoptotic bodies. To pellet and collect large oncosomes (LOs), the supernatant was transferred into a new centrifuge tube and centrifuged at 8,000×g for 30 min to pellet LOs, which was resuspended in a large volume of phosphate-buffered saline (PBS) following ultracentrifugation at 8,000×g for 30 min to precipitate LOs for further qNano analysis using NP2000nm nanopore. To remove any LOs contamination, the remaining supernatant was first centrifuged at 10,000×g for 30 min and transferred into a new centrifuge tube for further centrifuging 40 min at 15,000×g, then the pellet was resuspended in a large volume of phosphate-buffered saline (PBS) following ultracentrifugation at 15,000×g for 40 min to precipitate microvesicles (MVs) for further qNano analysis using NP800nm nanopore. To remove any remaining MVs, the media supernatant from the first 15,000×g step was passed through a 0.22mm pore polyethersulfone (PES) filter (Millipore). This supernatant (pre-cleared medium) was next subjected to ultracentrifugation at 120,000×g for 4 h in a SW32 Ti Rotor Swinging Bucket rotor (Beckman Coulter, Fullerton, CA) to sediment small extracellular vesicles (sEVs) for further qNano analysis using NP100nm nanopore. The crude sEV pellet was resuspended in a large volume of PBS followed by ultracentrifugation at 120,000×g for 4 h to wash the sample. At no time during the process were samples subjected to temperatures below 4°C.

187-1 Loading into LOs. For 187-1 loading into LOs, purified LOs (~10^8 LOs) were first mixed with 50μM 187-1 or Wiskostatin in 1 mL PBS. After incubation at 22°C for 60 minutes, the mixture was subjected to centrifugation for 30 minutes at 10,000×g to pellet the

LOs. The supernatant containing unbound 187-1 was removed, and the LOs/187-1 pellet was washed by suspending it in DPBS and pelleting it again at 10,000 × g for 30 minutes. Based on the TEM, the vesicles were determined to be LOs.

Immunoblotting analysis (IB). IB was performed according to a standard protocol with the following antibodies: anti-VAPA (ab225890), anti-N-WASP (ab126626), anti-ARP2 (ab128934), anti-ARP3 (ab181164), anti-G-actin (ab123034)), anti-ANXA1 (ab214486), anti-alpha V (ab179475), anti-CD9 (ab236630), anti-Alix (ab275377), anti-CD63 (ab271286), anti-H2A (ab177308), anti-HA (ab9110), anti-Myc (ab32072), anti-Na^+/K^+-ATPase (ab58475) antibodies were purchased from Abcam (Cambridge, MA). Anti-CK18 (CST #4548) antibody were purchased from Cell Signaling technology (Danvers, MA). Anti-Flag (F3165) and anti-HA (SAB1306169) antibodies purchased from Sigma-Aldrich (St. Louis, MO).

Immunofluorescence (IF) staining. The osteoclast precursors cells were placed in 24-well clusters containing glass coverslips (Thermo Fisher Scientific) for 24h and were treated with extracellular vesicles and non-extracellular vesicles from conditioned media (CM) of HCC cells for 6 days. Cells were rinsed briefly with PBS and fixed in 4% (w/v) paraformaldehyde in PBS for 20 min at 37 °C. Aspirate fixation solution and wash cells 2-3 times in PBS. And followed by the antibody: anti-VAPA (ab244312, 1:200), anti-N-WASP (ab126626, 1:300), anti-ARP2 (ab49674, 1:200), anti-ARP3 (ab181164, 1:50), anti-FLAG (Sigma MAB3118, 1:100) antibody. The secondary antibody was goat anti-rabbit IgG (H + L) conjugated with Alexa Fluor 488 (Thermo Fisher Scientific), or added 1:1000 dilution of Phalloidin-iFluor 594 (ab176757) in 1% BSA at room temperature for 20-90 minutes. Rinse cells 2-3 times with PBS (5 min/wash). Then cells were mounted with Antifade Mountant with DAPI (Thermo Fisher Scientific). And extracellular vesicles followed by the antibody: anti-flag-488 (ab245892, 1:50), anti-VAPA-494 (NBP2-97848A594, 1:50) and anti-αV-integrin-647 (ab204684, 1:100) antibodies. Observe the cells and extracellular

vesicles at Ex/Em 493/517 nm and the images were captured using the AxioVision Rel.4.6 computerized image analysis system (Carl Zeiss, Jena, Germany).

Immunohistochemistry (IHC). IHC analysis was performed to determine altered protein expression in paraffin-embedded normal liver tissues, hepatocellular carcinoma tissues and bone metastasis tissues with anti-VAPA (abcam181067) antibody overnight at 4℃. The scores were determined by combining the proportion of positively-stained tumor cells and the intensity of staining. The scores given by the two independent pathologists were combined into a mean score for further comparative evaluation. Tumor cell proportions were scored as follows: 0, no positive tumor cells; 1, <10% positive tumor cells; 2, 10%–35% positive tumor cells; 3, 35%–75% positive tumor cells; 4, >75% positive tumor cells. Staining intensity was graded according to the following standard: 1, no staining; 2, weak staining (light yellow); 3, moderate staining (yellow brown); 4, strong staining (brown). The staining index (SI) was calculated as the product of the staining intensity score and the proportion of positive tumor cells. Using this method of assessment, we evaluated protein expression in normal liver tissues, hepatocellular carcinoma tissues and bone metastasis tissues by determining the SI, with possible scores of 0, 2, 3, 4, 6, 8, 9, 12, and 16. Samples with a SI ≥ 8 were determined as high expression and samples with a SI < 8 were determined as low expression. Cutoff values were determined on the basis of a measure of heterogeneity using the log-rank test with respect to overall survival.

Enzyme-linked immunosorbent assay (ELISA). The femur and tibial were dissected from mice, and flushed into 1ml PBS solution and immediately kept on ice. And then it was centrifuged at 300 g for 5 minutes to collect bone marrow supernatant that stored at -80°C. The levels of CXCL12, CCL2 and CCL4 in bone marrow supernatant in femur and tibial of mice were measured using mouse CXCL12 ELISA Kit (ab100741), mouse CCL2 ELISA Kit (sigma-aldrich, RAB0056), mouse CCL4 ELISA Kit (sigma-aldrich, RAB0829), respectively.

Serum samples were kept at room temperature for about 1.0 h to defrost completely before assays. The VAPA level in serum and the culture medium from HCC cells were measured using a VAPA enzyme-linked immunosorbent assay kits (OKCA01588, Aviva Systems Biology, San Diego, California, USA.), and analyzed according to the manufacturer's instructions. The levels of TGF-β, RANKL and OPG in culture medium were measured using mouse TGF-β ELISA Kit (ab119557), human RANKL ELISA Kit (ab213841), mouse RANKL ELISA Kit (ab100749), human OPG ELISA Kit (ab100617) and mouse OPG ELISA Kit (ab203365), respectively. Data were read with the SpectraMax i3x Multi-Mode Microplate Reader (Molecular Devices) at 450 nm, the concentrations of VAPA in the samples were determined by extrapolating from the standard curve created by plotting the absorbance of the standards versus corresponding concentrations.

Quantification mass spectrometry data analysis. The resulting original chromatograms of each component were processed by ProteinPilot software (V4.5) of SCIEX for database search, protein identification and relative quantitative analysis. All proteins with a false discovery rate (FDR) under 1% were selected. Tandem spectra were searched against the Homo Sapiens protein database. Protein identifications were based on grouping of the matched peptide from all samples. The protein ratios were calculated from the median of the unique peptides. In addition, all peptide ratios were standardized using the median protein ratio, which should be 1 after normalization. Proteins with P-values under 0.05 and fold changes of more than 2 or less than 0.5 were deemed significantly regulated. And there was a total of 61 dysregulated proteins, including 36 upregulated proteins and 25 downregulated proteins, in LOs from HCCLM3-BM4 cells compared with LOs from HCCLM3-parental cells as showed in Table S1, Supporting Information.

Visual Assay for actin nucleation. An actin binding protein biochem kit (BK013; Cytoskeleton, Denver, USA) was used to perform actin nucleation assay according to the

manufacturer's instructions. Alexa488-labeled monomeric actin was added to a final concentration of 1.5μM. DMSO or 50 μM 187-1 or 25 μM Wiskostatin was added, and 150 nM recombinant N-WASP protein (ab132277) and/or 500nM recombinant VAPA protein (ab93462) were added to initiate assembly. Reaction mixtures were pressed between cover glasses and imaged on a Zeiss LSM 880 Confocal Microscope. And the fluorescence was measured every ≈1 min for ≈20 min at room temperature.

Isolation and culture of osteoclast precursors. To isolate osteoclast precursors from adult mouse, total bone marrow is isolated from the tibia and femur bones of 6-8-week-old C57 mice. First, scissors are used to cut the hindlimbs from the dead mice while keeping the long bones intact. And then, paws and skin were removed from the limbs and placed in PBS for transport. The limbs are transferred to a sterile Petri dish and separated the femur and the tibia. The remaining soft tissue is scraped from the bones and the epiphyses is cut off to expose the bone marrow. the bone marrow was flushed out from each bone using the culture medium. The cell suspension is then centrifuged at 300×g for 5 min, the supernatant is discarded and the pellet is resuspended in 1 ml of culture medium. To obtain human osteoclasts, density centrifugation is used to isolate human peripheral blood mononuclear cells (PBMCs) from samples of fresh peripheral/venous blood. These samples have been collected into EDTA coated anticoagulant vessels. First, dilute 10 ml of the blood sample with 10 ml of warm, sterile PBS. Then take 50 ml tube and pipet in 10 ml of Lymphoprep mixture. Slowly add the 20 ml of diluted blood sample onto the Lymphoprep using the side of the tube. The blood should form a layer on top of the Lymphoprep. Centrifuge the 50 ml tube at 800×g for 30 min with the brake off. This will separate the solution into three layers, of which the small middle layer contains the PBMCs. Carefully collect this layer and transfer it into a clean 50 ml tube. Add PBS until there is a total volume of 20 ml and resuspend the cells to wash them. Centrifuge the tubes at 300×g for 3 min and discard the

supernatant to remove any traces of Lymphoprep. Resuspend the cell pellet in 1 ml culture medium and estimate the cell number. The cells can be put straight into culture or aliquoted and stored in liquid nitrogen for use.

Osteoclastogenesis assay. The osteoclast precursor cells (1×10^5) were cultured on 24-well clusters containing glass coverslips (Thermo Fisher Scientific) and grown in the conditioned media (CM), and treated with DMSO or CK-666 (100μM), or LOs/187-1 (10^5 particles), or LOs/Wiskostatin (10^5 particles). Media were changed at every other day. Osteoclasts were counted on day 6. The osteoclasts cultured on plastic dishes were fixed with 4% paraformaldehyde/PBS and TRAP expression was stained with a commercial kit (387A-1KT; Sigma-Aldrich). Osteoclasts were defined as TRAP-positive multinucleated cells containing more than three nuclei.

Preparation of demineralized bone matrix. As described previously report[1], the mineralized bone slices were immersed in 1.2 N hydrochloric acid (HCl) solution to remove minerals from the bone matrix. Before use, the demineralized bone matrix was sterilized with 75% ethanol for 30 min and then washed with phosphate-buffered saline (PBS) three times with 10-min intervals.

Isolation of mesenchymal stem cells from bone marrow. MSCs were prepared from mouse bone marrow as described previously report [2]. In brief, 3-weeks -old male C57BL/6J mice were euthanized with an overdose of pentobarbital sodium (90 mg/kg) and femurs and tibiae were excised. And the whole bone marrow was flushed from the tibia and femur. MSCs preferentially attached to the polystyrene surface and were further purified by flow cytometry. MSCs were identified to express CD29 but not MHC class II antigens, costimulatory molecules CD80 and CD86 by flow cytometry. Isolated MSCs were cultured in osteoblast differentiation medium composed of α-MEM supplemented with 1% PS, 10%

FBS, 10 mM β-glycerophosphate, 200 μM l-ascorbic acid and 300 ng/ml BMP2.

Alizarin red mineral staining. Mature osteoblasts were cultured on demineralized bone matrix for up to 10 days with osteogenic differentiation medium. At the end of the experiment, cells were fixed with 4% formaldehyde for 5 min, washed three times with double distilled water, and stained with alizarin red S (Sigma-A5533) for 15 min and washed with double distilled water until the washing solution appeared clear. Mineral stained with alizarin red S was imaged with an CTL-ImmunoSpot analyzer (S6 ULTRA). Deposited mineral was quantified by solubilizing alizarin red in 10% acetic acid for 2 hour and measuring the absorbance of the solution at 405 nm with the SpectraMax i3x Multi-Mode Microplate Reader (Molecular Devices).

Isolation of hematopoietic stem cells from bone marrow. HSCs were isolated from mouse bone marrow using a modification of the method described by Shanmugam Muruganandan et al[3]. Briefly, 2-month-old male C57BL/6J mice were euthanized with an overdose of pentobarbital sodium (90 mg/kg) and femurs and tibiae were excised. The marrow was flushed with HSC medium (α-MEM containing 1% l-glutamine, 10% fetal bovine serum, and 1% PS) and passed by using a 26-gauge needle and then the cell suspension filtered through a 70 μm pore size cell strainer (BIOLOGIX, catalog number 15-1070-01). Lin^{-} $Sca1^{+}$ hematopoietic cells were isolated by sequential depletion of lineage positive (Lin^{+}) cells (MagCellect Mouse Hematopoietic Cell Lineage Depletion Kit, R&D Systems, catalog number MAGM209) and isolation of Sca-1-positive stem cells (Mouse $Sca\text{-}1^{+}$ Stem Cells kit, R&D Systems, Minneapolis, MN). The resulting Lin^{-} $Sca1^{+}$ cells were stained with 1 μg/ml Alexa Fluor® 594-conjugated rat anti-mouse c-kit (CD117) (R&D Systems, catalog number FAB1356T) and 1 μg/μl Alexa Fluor® 488-conjugated anti-mouse CD34 antibodies (Abcam, catalog number ab195013), and sorted by fluorescence-activated cell sorting to obtain Lin^{-} $Sca1^{+}$ $c\text{-}kit^{+}$ $CD34^{+}$ pure HSCs. Then isolated HSCs were seeded

on demineralized bone matrix, and demineralized bone matrix with osteoblasts with osteoclast differentiation medium composed of α-MEM supplemented with 10% FBS, 1% PS, and M-CSF (30 ng/ml). Osteoclast differentiation medium was replaced every 3 days.

Bone resorption pit assay. To study the ability of osteoclasts to form resorption pits on bone slices (IDS PLC, Boldon, UK; catalog number: DT-1BON1000-96), osteoclast precursor cells (1×10^5) were seeded onto the bone slices and cultured for 9 days in the presence of either extracellular vesicles from HCC cells conditioned media (CM) alone or together with DMSO or LOs/187-1 (10^5 particles), or LOs/Wiskostatin (10^5 particles). Media were changed at every other day. After 9 days, bone slices were fixed by 2.5% glutaraldehyde, then cells were removed by mechanical agitation and sonication. Resorption lacunae were visualized by scanning electron microscopy (SEM). Three fields were randomly selected for each bone slice for further analysis. Pit areas were quantified using ImageJ software.

Negative stain transmission electron microscopy (TEM). Extracellular vesicles and non-extracellular vesicles samples were then deposited on formvar carbon-coated grids (Electron Microscopy Sciences, Hatfield, PA) for 1 min followed by negative staining with 2% uranyl acetate for 30s at RT. Imaging was performed on a Philips/FEI T-12 Transmission Electron Microscope. Cells samples were fixed with 2% glutaraldehyde, 2% paraformaldehyde and 0.1 M PBS (pH 7.4) at RT for 15 min. The specimen was further fixed with 2% glutaraldehyde and 0.1 M PBS (pH 7.4) at 4°C. The cells were postfixed with OsO4, dehydrated and embedded in resin. Sections (70 nm thick) were obtained by cutting parallel to the substratum. Sectioning was performed between 0.5 and 0.6 mm above the substratum. The sections were stained with uranyl acetate and lead citrate. Photographs were taken on JEM-1400EX electron microscope (JOEL, Ltd, Japan).

Field emission scanning electron microscopy (FESEM) of the cytoskeleton of cultured

cells. Sample preparation for SEM was performed as described[4]. Two hours before sample preparation, whole cells were plated onto 24 mm glass coverslips. Immediately before fixation, the coverslips were washed three times with culture medium without serum and transferred to cytoskeleton buffer (50 mM imidazole, 50 mM KCl, 0.5 mM MgCl2, 0.1 mM EDTA, 1 mM EGTA, pH 6.8) containing 0.5% Triton-X and 0.25% glutaraldehyde for 5 min. This was followed by a second extraction with 2% Triton-X and 1% CHAPS in cytoskeleton buffer for 5 min before washing the coverslips in cytoskeleton buffer three times. The remainder of the protocol was identical to the originally described procedure[2]. The cells were dehydrated with serial ethanol dilutions, dried in a critical point dryer, coated with 5-6 nm platinum-palladium and imaged using the Zeiss/Bruker Field Emission Scanning Electron Microscope (Gemini500). All samples were prepared in duplicate and images from three independent experiments were acquired. Similar phenotypes were observed in each independent experiment and images of at least eight different cells were acquired for each experimental condition.

Cell growth assay. Cell growth was examined by 3-(4, 5-Dimethylthiazol-2-yl)-2, 5-diphenyltetrazolium bromide (MTT) assay. Briefly, cells were seeded at the density of 8000 cells/well into 96-well plate. At the time of assay, 0.5 mg/mL MTT in basic medium was added to each well and incubated for 4 h. After removing MTT, dimethyl sulfoxide (DMSO) was added and mixed vigorously. Absorbance was measured at 490 nm using the SpectraMax i3x Multi-Mode Microplate Reader (Molecular Devices).

Alkaline phosphatase (ALP) staining. Fourteen days after osteogenic induction, a TRAP/ALP Stain Kit (Wako, Richmond, VA, USA; 294-67001) was used to perform alkaline phosphatase (ALP) staining. The cells cultured in 24-well plates were rinsed three times by PBS and fixated for 30 min using pre-cold fixative. Next, ALP substrate solution was added and cells were cultivated (room temperature, 15-45min) in a darkroom. After that, cells with

ddH2O were rinsed and filmed under an optical microscope. The absorbance at 405 nm of each well was measured with a microplate reader according to the manufacturer's instruction.

Micro-CT (μCT) analysis. The hind limbs were removed from euthanized mice and fixed in either 4% paraformaldehyde solution or periodate-lysine-paraformaldehyde fixative. Fixed hind limbs were dissected free of tissue and scanned on a micro-CT scanner (SIEMENS, Munich, Germany). A standard trabecular volume of interest was chosen starting 0.1 mm from the growth plate and included all trabeculae in a 1 mm^3 region of bone. Trabecular volume and number were assessed in this region. Osteolytic lesions were measured through 360° view of the bone on a three-dimensional model in a 3 mm length of cortical bone, starting at the growth plate.

***In vivo* quantification of osteoclast number.** Hind limbs were fixed in paraformaldehyde solution (4%), decalcified in 14.3% EDTA for 4 days at 37 °C with daily changes of EDTA, then embedded in paraffin wax. Sections were used by H&E stained with Mayer's hematoxylin solution, stained with TRAP (a TRAP kit, 387A-1KT; Sigma-Aldrich) according to manufacturer's protocols. The numbers of $TRAP^+$-osteoclasts were determined on a 3 mm length of endocortical surface and viewed on an optical microscope (Olympus, DP72, Tokyo, Japan).

References

[1] Y. Park, E. Cheong, J. G. Kwak, R. Carpenter, J. H. Shim, J. Lee, *Sci Adv* **2021**, 7.

[2] a) S. Huang, L. Xu, Y. Sun, T. Wu, K. Wang, G. Li, *J Orthop Translat* **2015**, 3, 26; b) S. Xu, A. De Becker, B. Van Camp, K. Vanderkerken, I. Van Riet, *J Biomed Biotechnol* **2010**, 2010, 105940.

[3] S. Muruganandan, H. J. Dranse, J. L. Rourke, N. M. McMullen, C. J. Sinal, *Stem Cells* **2013**, 31.

[4] a) L. Cao, A. Yonis, M. Vaghela, E. H. Barriga, P. Chugh, M. B. Smith, J. Maufront, G. Lavoie, A. Meant, E. Ferber, M. Bovellan, A. Alberts, A. Bertin, R. Mayor, E. K. Paluch, P. P. Roux, A. Jegou, G. Romet-Lemonne, G. Charras, *Nat Cell Biol* **2020**, 22, 803; b) T. M. Svitkina, G. G. Borisy, *Methods Enzymol* **1998**, 298, 570.

Supplementary Tables

Table S1: Proteins in protein profiling of LOs from HCCLM3-BM4 vs. LOs from HCCLM3 cells

Uniprot ID Protein name	Description	HCCLM3	HCCLM3	HCCLM3	HCCLM3 -BM4	HCCLM3 -BM4	HCCLM3 -BM4	Log2 FoldChange	pvalue
Q9P0L0|VAPA_HUMAN	Vesicle-associated membrane protein-associated protein A OS=Homo sapiens OX=9606 GN=VAPA PE=1 SV=3	0.278450	0.343732	0.446351	1.362502	1.430850	1.598897	2.039327	0.0002
P05556|ITB1_HUMAN	Integrin beta-1 OS=Homo sapiens OX=9606 GN=ITGB1 PE=1 SV=2	0.443880	0.199802	0.423764	1.303779	1.144623	1.144620	1.751035	0.0009
Q92917|GPKOW_HUMAN	G-patch domain and KOW motifs-containing protein OS=Homo sapiens OX=9606 GN=GPKOW PE=1 SV=2	0.146357	0.188096	0.074784	0.450654	0.486335	0.386459	1.693290	0.0023
Q6UVK1|CSPG4_HUMAN	Chondroitin sulfate proteoglycan 4 OS=Homo sapiens OX=9606 GN=CSPG4 PE=1 SV=2	0.392894	0.271156	0.404679	1.288711	1.073969	1.083787	1.689223	0.0006
P12235|ADT1_HUMAN	ADP/ATP translocase 1 OS=Homo sapiens OX=9606 GN=SLC25A4 PE=1 SV=4	0.125526	0.272185	0.137005	0.460335	0.544152	0.710807	1.681609	0.0108
P26006|ITA3_HUMAN	Integrin alpha-3 OS=Homo sapiens OX=9606 GN=ITGA3 PE=1 SV=5	0.107955	0.168528	0.249671	0.434041	0.489403	0.685157	1.612249	0.0140
P46379|BAG6_HUMAN	Large proline-rich protein BAG6 OS=Homo sapiens OX=9606 GN=BAG6 PE=1 SV=2	0.393139	0.479578	0.198421	1.203856	0.991327	0.943390	1.550964	0.0040
P21796|VDAC1_HUMAN	Voltage-dependent anion-selective channel protein 1 OS=Homo sapiens OX=9606 GN=VDAC1 PE=1 SV=2	0.094758	0.199245	0.136655	0.345387	0.522425	0.386494	1.542273	0.0111

O75947|ATP5H_HUMAN	ATP synthase subunit d mitochondrial OS=Homo sapiens OX=9606 GN=ATP5PD PE=1 SV=3	0.324383	0.167331	0.077491	0.336141	0.586397	0.610877	1.429728	0.0474
P05362|ICAM1_HUMAN	Intercellular adhesion molecule 1 OS=Homo sapiens OX=9606 GN=ICAM1 PE=1 SV=2	0.235383	0.408634	0.421984	0.919665	1.104118	0.773908	1.392028	0.0069
Q9Y679|AUP1_HUMAN	Lipid droplet-regulating VLDL assembly factor AUP1 OS=Homo sapiens OX=9606 GN=AUP1 PE=1 SV=2	0.132459	0.073475	0.260769	0.365338	0.404836	0.422665	1.353825	0.0139
Q8TEA8|DTD1_HUMAN	D-aminoacyl-tRNA deacylase 1 OS=Homo sapiens OX=9606 GN=DTD1 PE=1 SV=2	0.342036	0.308254	0.411644	0.816761	0.999192	0.869367	1.338400	0.0010
P05141|ADT2_HUMAN	ADP/ATP translocase 2 OS=Homo sapiens OX=9606 GN=SLC25A5 PE=1 SV=7	0.150093	0.149607	0.090781	0.362137	0.347213	0.276204	1.335681	0.0039
O00193|SMAP_HUMAN	Small acidic protein OS=Homo sapiens OX=9606 GN=SMAP PE=1 SV=1	0.384257	0.370962	0.317023	1.001406	0.944326	0.758195	1.334426	0.0020
Q9Y6A5|TACC3_HUMAN	Transforming acidic coiled-coil-containing protein 3 OS=Homo sapiens OX=9606 GN=TACC3 PE=1 SV=1	0.275145	0.400899	0.386748	0.895105	0.985403	0.794718	1.331802	0.0014
P45880|VDAC2_HUMAN	Voltage-dependent anion-selective channel protein 2 OS=Homo sapiens OX=9606 GN=VDAC2 PE=1 SV=2	0.415036	0.225361	0.432073	0.882500	0.921475	0.884014	1.325591	0.0013
P21333|FLNA_HUMAN	Filamin-A OS=Homo sapiens OX=9606 GN=FLNA PE=1 SV=4	0.175809	0.171655	0.054978	0.285099	0.354603	0.367836	1.323980	0.0129

Q13263|TIF1B_HUMAN	Transcription intermediary factor 1-beta OS=Homo sapiens OX=9606 GN=TRIM28 PE=1 SV=5	0.542943	0.307440	0.211768	0.936654	0.877299	0.839916	1.321107	0.0066
Q14677|EPN4_HUMAN	Clathrin interactor 1 OS=Homo sapiens OX=9606 GN=CLINT1 PE=1 SV=1	0.321818	0.300403	0.439784	0.879722	0.844978	0.892822	1.301411	0.0003
Q9H9S4|CB39L_HUMAN	Calcium-binding protein 39-like OS=Homo sapiens OX=9606 GN=CAB39L PE=1 SV=3	0.200073	0.123622	0.109849	0.383639	0.391027	0.286626	1.291573	0.0088
Q8WY22|BRI3B_HUMAN	BRI3-binding protein OS=Homo sapiens OX=9606 GN=BRI3BP PE=1 SV=1	0.167308	0.187139	0.161872	0.390535	0.466702	0.406208	1.291030	0.0005
P12236|ADT3_HUMAN	ADP/ATP translocase 3 OS=Homo sapiens OX=9606 GN=SLC25A6 PE=1 SV=4	0.403411	0.411024	0.250099	0.982319	0.837400	0.774435	1.285042	0.0032
P62330|ARF6_HUMAN	ADP-ribosylation factor 6 OS=Homo sapiens OX=9606 GN=ARF6 PE=1 SV=2	0.391128	0.436990	0.234053	0.678537	0.892803	0.970471	1.258841	0.0099
P42704|LPPRC_HUMAN	Leucine-rich PPR motif-containing protein mitochondrial OS=Homo sapiens OX=9606 GN=LRPPRC PE=1 SV=3	0.405790	0.341037	0.318081	0.900530	0.937682	0.703129	1.254861	0.0031
P25705|ATPA_HUMAN	ATP synthase subunit alpha mitochondrial OS=Homo sapiens OX=9606 GN=ATP5F1A PE=1 SV=1	0.422649	0.294056	0.347687	0.849041	0.871449	0.799715	1.243512	0.0003
O00410|IPO5_HUMAN	Importin-5 OS=Homo sapiens OX=9606 GN=IPO5 PE=1 SV=4	0.276002	0.379953	0.418167	0.891966	0.732766	0.879173	1.221022	0.0020
Q07065|CKAP4_HUMAN	Cytoskeleton-associated protein 4 OS=Homo sapiens OX=9606 GN=CKAP4 PE=1 SV=2	0.309786	0.462718	0.296297	0.826955	0.856054	0.786525	1.208245	0.0012

O15173|PGRC2_HUMAN	Membrane-associated progesterone receptor component 2 OS=Homo sapiens OX=9606 GN=PGRMC2 PE=1 SV=1	0.373550	0.340818	0.358778	0.921181	0.807362	0.740920	1.202352	0.0010
Q13409|DC1I2_HUMAN	Cytoplasmic dynein 1 intermediate chain 2 OS=Homo sapiens OX=9606 GN=DYNC1I2 PE=1 SV=3	0.143351	0.135746	0.110956	0.304452	0.332100	0.248218	1.181630	0.0034
O43399|TPD54_HUMAN	Tumor protein D54 OS=Homo sapiens OX=9606 GN=TPD52L2 PE=1 SV=2	0.125714	0.074696	0.217113	0.332100	0.366867	0.243350	1.174360	0.0346
P13693|TCTP_HUMAN	Translationally-controlled tumor protein OS=Homo sapiens OX=9606 GN=TPT1 PE=1 SV=1	0.326059	0.311282	0.428887	0.858431	0.748110	0.784961	1.165401	0.0009
P00966|ASSY_HUMAN	Argininosuccinate synthase OS=Homo sapiens OX=9606 GN=ASS1 PE=1 SV=2	0.342917	0.161533	0.216837	0.504631	0.630008	0.479898	1.162476	0.0138
P07948|LYN_HUMAN	Tyrosine-protein kinase Lyn OS=Homo sapiens OX=9606 GN=LYN PE=1 SV=3	0.296866	0.303459	0.469863	0.852915	0.852305	0.690023	1.162307	0.0049
P07686|HEXB_HUMAN	Beta-hexosaminidase subunit beta OS=Homo sapiens OX=9606 GN=HEXB PE=1 SV=3	0.256319	0.061257	0.179450	0.376634	0.408438	0.311799	1.142001	0.0345
P16422|EPCAM_HUMAN	Epithelial cell adhesion molecule OS=Homo sapiens OX=9606 GN=EPCAM PE=1 SV=2	0.332810	0.554849	0.179629	0.771805	0.772960	0.739457	1.097753	0.0207
P56385|ATP5I_HUMAN	ATP synthase subunite mitochondrial OS=Homo sapiens OX=9606 GN=ATP5ME PE=1 SV=2	0.096692	0.217118	0.161873	0.354011	0.273763	0.337059	1.020280	0.0185
Q92973|TNPO1_HUMAN	Transportin-1 OS=Homo sapiens OX=9606 GN=TNPO1 PE=1 SV=2	0.771723	0.769418	0.897486	0.309006	0.435707	0.457435	-1.020454	0.0028

P07195|LDHB_HUMAN	L-lactate dehydrogenase B chain OS=Homo sapiens OX=9606 GN=LDHB PE=1 SV=2	0.779241	0.817685	0.475371	0.424929	0.370617	0.195130	-1.064745	0.0486
Q86VM9|ZCH18_HUMAN	Zinc finger CCCH domain-containing protein 18 OS=Homo sapiens OX=9606 GN=ZC3H18 PE=1 SV=2	0.869711	0.743194	0.465117	0.397825	0.292654	0.298582	-1.071079	0.0432
Q9BRQ6|MIC25_HUMAN	MICOS complex subunit MIC25 OS=Homo sapiens OX=9606 GN=CHCHD6 PE=1 SV=1	0.742475	0.791601	0.600300	0.318763	0.443757	0.227252	-1.108647	0.0109
O95248|MTMR5_HUMAN	Myotubularin-related protein 5 OS=Homo sapiens OX=9606 GN=SBF1 PE=1 SV=4	0.853755	0.861942	0.881081	0.465622	0.322865	0.364945	-1.170789	0.0004
Q8IYB5|SMAP1_HUMAN	Stromal membrane-associated protein 1 OS=Homo sapiens OX=9606 GN=SMAP1 PE=1 SV=2	0.761894	0.826167	0.531482	0.391177	0.369314	0.177927	-1.175450	0.0247
Q96G28|CFA36_HUMAN	Cilia- and flagella-associated protein 36 OS=Homo sapiens OX=9606 GN=CFAP36 PE=1 SV=2	0.778662	0.942208	0.841222	0.485359	0.301104	0.319128	-1.212505	0.0030
Q8NCH0|CHSTE_HUMAN	Carbohydrate sulfotransferase 14 OS=Homo sapiens OX=9606 GN=CHST14 PE=1 SV=2	0.751894	0.744731	1.085986	0.250234	0.325213	0.511521	-1.248521	0.0219
P29401|TKT_HUMAN	Transketolase OS=Homo sapiens OX=9606 GN=TKT PE=1 SV=3	0.812522	0.864771	0.802945	0.523129	0.140588	0.371587	-1.260425	0.0130
B4DS77|SHSA9_HUMAN	Protein shisa-9 OS=Homo sapiens OX=9606 GN=SHISA9 PE=2 SV=3	0.646572	0.576539	0.949579	0.192680	0.480613	0.228152	-1.269171	0.0440
O95707|RPP29_HUMAN	Ribonuclease P protein subunit p29 OS=Homo sapiens OX=9606 GN=POP4 PE=1 SV=2	0.807214	0.756896	0.896445	0.355690	0.337898	0.265938	-1.358590	0.0005

Q9P0J1|PDP1_HUMAN	[Pyruvate dehydrogenase [acetyl-transferring]]-phosphatase1 mitochondrial OS=Homo sapiens OX=9606 GN=PDP1 PE=1 SV=3	0.878904	1.169201	0.619640	0.369870	0.136828	0.527178	-1.367557	0.0492
Q14676|MDC1_HUMAN	Mediator of DNA damage checkpoint protein 1 OS=Homo sapiens OX=9606 GN=MDC1 PE=1 SV=3	0.939875	0.697319	0.911521	0.383438	0.230038	0.360460	-1.387869	0.0043
P30041|PRDX6_HUMAN	Peroxiredoxin-6 OS=Homo sapiens OX=9606 GN=PRDX6 PE=1 SV=3	0.604652	0.855262	0.662556	0.262116	0.260384	0.282512	-1.398662	0.0045
Q14738|2A5D_HUMAN	Serine/threonine-protein phosphatase 2A 56 kDa regulatory subunit delta isoform OS=Homo sapiens OX=9606 GN=PPP2R5D PE=1 SV=1	0.635686	0.639184	0.717594	0.284495	0.250360	0.218209	-1.403709	0.0002
Q9H061|T126A_HUMAN	Transmembrane protein 126A OS=Homo sapiens OX=9606 GN=TMEM126A PE=1 SV=1	0.819296	1.003422	0.746772	0.185924	0.264870	0.509885	-1.419356	0.0124
O95478|NSA2_HUMAN	Ribosome biogenesis protein NSA2 homolog OS=Homo sapiens OX=9606 GN=NSA2 PE=1 SV=1	0.781018	0.798161	0.964074	0.427253	0.208209	0.290912	-1.457008	0.0034
Q9H9A6|LRC40_HUMAN	Leucine-rich repeat-containing protein 40 OS=Homo sapiens OX=9606 GN=LRRC40 PE=1 SV=1	0.925224	0.909956	0.733601	0.234449	0.399466	0.266520	-1.512389	0.0022
O75531|BAF_HUMAN	Barrier-to-autointegration factor OS=Homo sapiens OX=9606 GN=BANF1 PE=1 SV=1	0.996316	0.807751	0.731995	0.202729	0.252902	0.432236	-1.514175	0.0064
Q9BW19|KIFC1_HUMAN	Kinesin-like protein KIFC1 OS=Homo sapiens OX=9606 GN=KIFC1 PE=1 SV=2	0.817766	0.686953	0.972901	0.215274	0.195242	0.430829	-1.558184	0.0082

Q6XZF7|DNMBP_HUMAN	Dynamin-binding protein OS=Homo sapiens OX=9606 GN=DNMBP PE=1 SV=1	0.906183	1.063896	0.902859	0.398163	0.227258	0.306144	-1.624798	0.0009
Q96AG4|LRC59_HUMAN	Leucine-rich repeat-containing protein 59 OS=Homo sapiens OX=9606 GN=LRRC59 PE=1 SV=1	1.069177	1.003706	0.982273	0.195212	0.353270	0.360271	-1.749287	0.0003
Q15642|CIP4_HUMAN	Cdc42-interacting protein 4 OS=Homo sapiens OX=9606 GN=TRIP10 PE=1 SV=3	0.934916	0.728143	0.903178	0.173153	0.310866	0.240518	-1.824523	0.0013
P35232|PHB_HUMAN	Prohibitin OS=Homo sapiens OX=9606 GN=PHB PE=1 SV=1	0.722199	1.029965	0.582613	0.155274	0.297651	0.187353	-1.866515	0.0153
P30048|PRDX3_HUMAN	Thioredoxin-dependent peroxide reductase mitochondrial OS=Homo sapiens OX=9606 GN=PRDX3 PE=1 SV=3	0.519636	0.946843	0.780160	0.119503	0.066639	0.373395	-2.005463	0.0228

Table S2. Clinicopathological Characteristics of Clinical Samples and Expression of VAPA in HCC

Characteristics	No. patients	(%)
**Age (years)**		
≤ 60	201	0.56
> 60	157	0.43
**Gender**		
Male	303	0.85
Female	55	0.15
**HBsAg**		
positive	314	0.882
negative	44	0.12
**HCV-Ab**		
positive	11	0.03
negative	347	0.97
**Clinical stage**		
I-II	252	0.70
III-IV	106	0.30
**Tumor size**		
> 5 cm	174	0.49
≤ 5 cm	184	0.51
**Intrahepatic control**		
Yes	261	0.73
No	97	0.27
**Bone metastasis**		
Yes	26	0.07
No	332	0.93
**Other extrahepatic metastasis**		
Yes	31	0.09
No	327	0.91

Continued

Table S2 (Continued**)**

**Characteristics**	**No. patients**	**(%)**
**Vital status (at follow-up)**		
Alive	233	0.65
Death due to liver cancer cause	125	0.35
**Expression of VAPA**		
Low expression	317	0.89
High expression	41	0.11

HBsAg: hepatitis B surface antigen, HCV-Ab: hepatitis C virus antibody, VAPA: VAMP associated protein A

Table S3. Correlation between VAPA expression and clinicopathologic characteristics of HCC Patient

Characteristics	VAPA Protein level		Chi-square test
	Low	High	*P* value
**Age (years)**			
≤ 60	182	19	0.179
> 60	135	22	
**Gender**			
Male	268	35	0.337
Female	49	6	
**HBsAg**			
positive	277	37	0.599
negative	40	4	
**HCV-Ab**			
positive	10	1	0.803
negative	307	40	
**Clinical stage**			
I-II	238	14	< 0.001
III-IV	79	27	
**Tumor size**			
> 5 cm	155	19	0.758
≤ 5 cm	162	22	
**Intrahepatic control**			
Yes	231	30	0.968
No	86	11	
**Bone metastasis**			
Yes	0	26	< 0.001
No	317	15	
**Other extrahepatic metastasis**			
Yes	29	2	0.36
No	288	39	
**Vital status (at follow-up)**			
Alive	223	10	< 0.001
Death	94	31	

HBsAg: hepatitis B surface antigen, HCV-Ab: hepatitis C virus antibody, VAPA: VAMP associated protein A

Table S4. Univariate and Multivariate Analyses of Various Prognostic Parameters in HCC Patients by Cox-regression Analysis

	**Univariate analysis**			**Multivariate analysis**		
	C. OR	95% CI	P-value	A. OR	95% CI	P-value
Clinical stage	36.697	21.947-61.358	< 0.001	41.246	23.306-72.995	< 0.001
Bone metastasis	9.323	5.947-14.615	< 0.001	0.294	0.101-0.857	0.025
Exp. of VAPA	4.282	2.842-6.450	< 0.001	5.803	2.167-15.537	< 0.001

Exp.: Expression; Abbreviations: C. OR, crude odds ratio; CI, confidence interval; A. OR, adjusted odds ratio;

Table S5. Primers and Oligonucleotides used in this study.

**Primer used for subcloning and plasmid construction**	
VAPA-myc-up	gtgtcgtgaggattgggatccGCCATGGCGTCCGCCTCAGGGGCCAT
VAPA-myc-down	cttcatatgttcgaagaattcCTACAGATCCTCTTCAGAGATGAGTTTC
VAPA-MSP-MYC-up	gtgtcgtgaggattgggatccGCCATGTCCTGGTCCTCGATCCGCCC
VAPA-MSP-MYC-down	cttcatatgttcgaagaattcCTACAGATCCTCTTCAGAGATGAGTTTC
VAPA-CC-MYC-up	gtgtcgtgaggattgggatccGCCATGAAGGATGACCCCAGGGGACT
VAPA-CC-MYC-down	cttcatatgttcgaagaattcCTACAGATCCTCTTCAGAGATGAGTTTC
VAPA-TM-MYC-up	gtgtcgtgaggattgggatccGCCATGCAGGGAGAAATGATGAAGCT
N-WASP-flag-up	gtgtcgtgaggattgggatccGCCATGAGCTCCGTCCAGCAGCAGCC
N-WASP-flag-down	cttcatatgttcgaagaattcTCACTTATCGTCGTCATCCTTGTAA
N-WASP-WH1-flag-up	gtgtcgtgaggattgggatccGCCATGCTCGGCAAGAAATGTGTGAC
N-WASP-WH1-flag-down	cttcatatgttcgaagaattcTCACTTATCGTCGTCATCCTTGTAA
N-WASP-B-flag-up	gtgtcgtgaggattgggatccGCCATGAGACGAGATCCCCCAAATGG
N-WASP-B-flag-down	cttcatatgttcgaagaattcTCACTTATCGTCGTCATCCTTGTAA
N-WASP-CRIB-Flag-up	gtgtcgtgaggattgggatccGCCATGAAGAGATTAACCAAGGCAGA
N-WASP-CRIB-Flag-down	cttcatatgttcgaagaattcTCACTTATCGTCGTCATCCTTGTAA
N-WASP-AI-Flag-up	gtgtcgtgaggattgggatccGCCATGAAAGTTATATATGACTTTAT
N-WASP-AI-Flag-down	cttcatatgttcgaagaattcTCACTTATCGTCGTCATCCTTGTAA
N-WASP-PRD-Flag-up	gtgtcgtgaggattgggatccGCCATGCCACCACCTCCACCACCATC
N-WASP-PRD-Flag-down	cttcatatgttcgaagaattcTCACTTATCGTCGTCATCCTTGTAA
N-WASP-VCA-flag-up	gtgtcgtgaggattgggatccGCCATGAACAAAGCAGCTCTTTTAGA
N-WASP-VCA-flag-down	cttcatatgttcgaagaattcTCACTTATCGTCGTCATCCTTGTAA
N-WASP-VCA-myc-up	gtgtcgtgaggattgggatccGCCATGCCTTCTGATGGGG
N-WASP-VCA-myc-down	cttcatatgttcgaagaattcTCACAGATCCTCTTCAGAGATGAGTT
N-WASP-ΔVCA-HA-up	gtgtcgtgaggattgggatccGCCATGAGCTCCGTCCAGC
N-WASP-ΔVCA-HA-down	cttcatatgttcgaagaattcTCAAGCGTAATCTGGAACATCG

Sequences mature sense of shRNA/siRNA	
VAPA shRNA#1	GATCCCCCAAGGAAACTAATGGAAGATTCAAGAGATCTTCCAT TAGTTTCCTTGTTTTTA
VAPA shRNA#2	GATCCCCCCTGATGAATTAATGGATTTTCAAGAGAAATCCATTA ATTCATCAGGTTTTTA
N-WASP siRNA#1	GACAGGGTATCCAACTAAT
N-WASP siRNA#2	AGATACACAGGGTATCCAA
Alpha-V shRNA#1	GATCCCCCCCAACTTCATTATAGATTTTTCAAGAGAAAATCTAT AATGAAGTTGG TTTTTA
Alpha-V shRNA#2	GATCCCCCAAATCTATAATGAAGTTGGTTCAAGAGACCAACTT CATTATAGATTT TTTTTA
